# Extending the Flory–Huggins Theory for Crystalline Multicomponent Mixtures

**DOI:** 10.1021/acs.macromol.5c02297

**Published:** 2026-02-11

**Authors:** Maxime Siber, Olivier J. J. Ronsin, Jens Harting

**Affiliations:** † Helmholtz Institute Erlangen-Nürnberg for Renewable Energy, 28334 Forschungszentrum Jülich, Fürther Straße 248, 90429 Nürnberg, Germany; ‡ Department of Chemical and Biological Engineering, Friedrich-Alexander-Universität Erlangen-Nürnberg, Fürther Straße 248, 90429 Nürnberg, Germany; § Department of Physics, Friedrich-Alexander-Universität Erlangen-Nürnberg, Fürther Straße 248, 90429 Nürnberg, Germany

## Abstract

The Flory–Huggins theory is a well-established lattice model that is commonly used to study the mixing of distinct chemical species. It can successfully predict phase separation phenomena in blends of incompatible materials. However, it is limited to amorphous mixtures, excluding systems where the phase segregation is shaped by the concurrent crystallization of one or several blend components. A generalization of the Flory–Huggins formalism is thus necessary to capture the coupling and the interplay of crystallization with amorphous demixing mechanisms, such as spinodal decomposition. This work, therefore, revolves around the derivation of a free energy model for multicomponent mixtures that encompasses the physics of both processes. It is detailed which concepts from the original Flory–Huggins theory are required to apprehend the presented developments and how the current framework is built upon them. Furthermore, additional discussion points address chemical potential calculations and selected examples of binary and ternary phase diagrams, thereby highlighting the variety of blend behaviors that can be represented.

## Graphical abstract

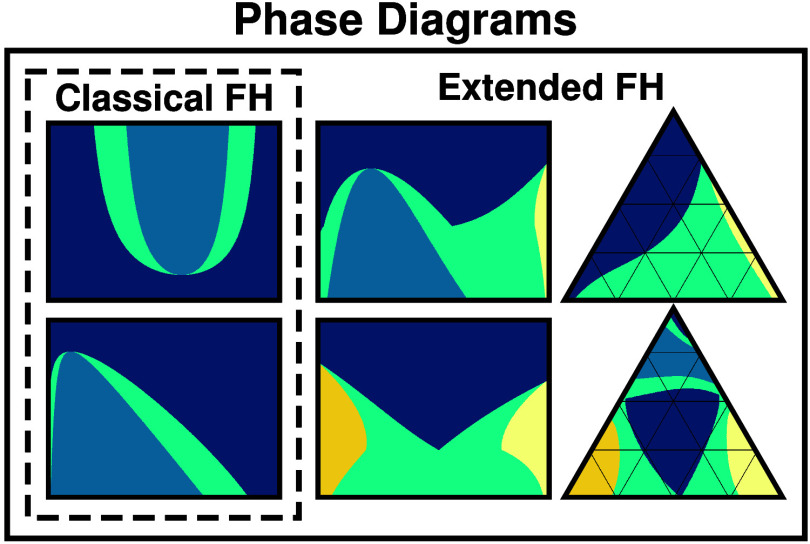

## Introduction

The Flory–Huggins theory^1,2^ provides a mathematical formalism that describes the thermodynamics of material mixtures. It relies on a virtual lattice representation, which allows to evaluate the spatial arrangements of chemical species of different sizes, such as polymers and small molecules, for instance. The model was first developed for binary systems, but generalizations for any amorphous blend, regardless of the number of components, are usually employed as well.^1,3–11^

Predictions from the Flory–Huggins framework were demonstrated to agree with qualitative observations from experiments.^1,12^ Especially, mixtures that exhibit miscibility gaps and are prone to spinodal decomposition behavior are accounted for. Quantitatively, the theoretical expectations from the Flory–Huggins free energy model are in line with measurements,^1,6,13–15^ even though corrections have to be implemented for material combinations where mixing interactions display complex dependencies on temperature, composition, and chemical structure.^6,10,16–20^

The theory finds applications in various research areas such as drug-polymer systems,^21–24^ organic electronics,^25–28^ and polymeric membrane manufacturing,^4,8,10,29^ for example. Recent efforts have been dedicated to determine analytical solutions for the binodal equilibrium compositions predicted by the model, so as to facilitate its usage.^30,31^ A remaining limitation of the treatment of mixing in the Flory–Huggins free energy framework is its restriction to amorphous components, while many materials can undergo crystallization phase transitions, even in the blend.

The objective of this work is therefore to derive an extended model that is based on the classical Flory–Huggins theory and captures crystallization phenomena. For this purpose, the proposed approach follows a generalized version of the mean-field approximation that is usually applied to the enthalpic mixing interactions in the fully amorphous case.^12^ Some introduced features are also inspired by previous publications by Matkar and Kyu^32,33^ where the Flory–Huggins formalism was augmented with elements from the Landau theory for phase transitions^34^ and Phase-Field modeling^11,35–37^ to obtain an expression for the free energy density of crystallization in binary mixtures. In addition, the common assumption that the latent heat release accompanying crystallization is linear with the degree of undercooling^38^ (as, for example, in the treatment of polymer crystallization by Hoffman and Lauritzen^39^) is used here as well.

Following this introduction, the present manuscript is divided into four successive sections: The first one contains an overview of the aspects of the original theory that subsequent developments are built upon. The second then details the derivation of the free energy formula for multicomponent blends with any number of crystalline constituents. The third discusses further chemical potential calculations and features a showcase study of phase diagrams generated from the model. Finally, the fourth section exposes the conclusions of this work.

## Free Energy in the Classical Flory–Huggins Framework

This section reviews core concepts from the classical Flory–Huggins theory. The purpose is not to detail the derivation of the fundamental model equations, as this can readily be found elsewhere in the literature,^1,12^ but rather to provide a reminder of the theoretical framework that the upcoming developments rely on. In this context, a material mixture is viewed as the arrangement of its different chemical constituents on a virtual lattice, hereafter referred to as the Flory–Huggins lattice (see Figure 1). As a result, each component can occupy one or several sites of the lattice, depending on its size compared to the reference volume of a lattice element. For convenience, the size of the smallest species in the blend is usually taken to determine the dimension of the Flory–Huggins lattice. In principle, the reference volume of a lattice element is, however, arbitrary. Despite having originally been built for binary polymer blends, the theory can be extended for amorphous mixtures with any number of components.^1,8,9,11,26^ For simplicity, the focus of this summary is restricted to mixtures involving two species only.

**Figure 1:**
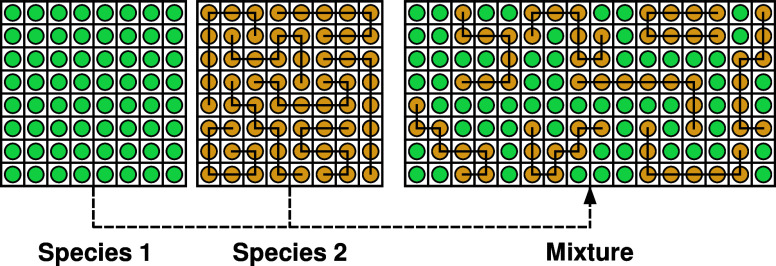
Schematic illustration of the mixing of a polymer solution on a two-dimensional Flory–Huggins lattice. The size proportions are *N*_2_ = 8 for the polymer (in yellow) to *N*_1_ = 1 for the solvent (in green).

The advantage of the Flory–Huggins approach lies in its relatively simple formulation for the system’s free energy change upon mixing Δ*G*, namely

1 $$\Delta G=\mathrm{kT}\left[{\mathrm{n̅}}_{1}\ln\left(\phi_{1}\right)+{\mathrm{n̅}}_{2}\ln\left(\phi_{2}\right)+{\mathrm{n̅}}_{0}\phi_{1}\phi_{2}\chi_{12}\right]$$

Here, *k* is the Boltzmann constant and *T* the temperature. *n̅*_0_ represents the total number of Flory–Huggins lattice sites. Analogously, *n̅*_1_ and *n̅*_2_ denote the number of particles for the first and the second species, respectively. Their overall volume fractions are then symbolized by ϕ_1_ and ϕ_2_. The terms involving the logarithms describe the free energy change due to the entropy increase upon ideal mixing and are consistent with the predictions from regular solution theory.^40–42^ Equation 1 slightly differs from the equation originally presented by Flory^1^ because it does not consider the Flory–Huggins lattice site volume to be necessarily equal to volume of the smallest species in the blend, as discussed in the Supporting Information (SI-A). Note also that Δ*G* conventionally refers to the Gibbs free energy. The formula for the Helmholtz counterpart (Δ*F*) is nonetheless equivalent, the theory assuming that mixing occurs under constant volume and pressure conditions.

In order to capture material mixtures that deviate from ideal mixing behavior, the Flory–Huggins model also includes a contribution that is controlled by the parameter χ_12_. This parameter accounts for attractive interactions arising between pairs of different species that occupy nearest-neighbor sites on the Flory–Huggins lattice^1,12^ (χ_12_ > 0 meaning that like species attract each other more than unlike ones, and vice versa for χ_12_ < 0). It is defined as

2 $$\chi_{12}≔\frac{z}{\mathrm{kT}}\Delta w=\frac{z}{\mathrm{kT}}\left[w_{12}-\frac{1}{2}\left(w_{11}+w_{22}\right)\right]$$

where Δ*w* is the energy gain per nearest-neighbor contact. Δ*w* originates from the interaction energy of a contact between both species *w*_12_, which, upon mixing, replaces the interaction energy of a component with itself (*w*_11_ or *w*_22_).^1,43^ In addition, *z* is the so-called coordination number, that is the number of nearest neighbors to a site of the lattice. In the most general case, Δ*w* is assumed to be of the same nature as a free energy, hence being decomposable in an enthalpy (or internal energy) and an entropy part. Empirically, the variation of χ_12_ with temperature is indeed found to obey the following relationship in many situations:^12^

3 $$\chi_{12}=A+\frac{B}{T}$$

*A* and *B* are the constant coefficients of the entropic and enthalpic contributions, respectively. Nevertheless, cases exist where this linear form in 1/*T* is not fitting the measurements.^19^ Additionally, χ_12_ is overall expected to be composition-dependent,^12,16,18,19^ even though this is not directly addressed within the framework of the classical Flory–Huggins theory. It has to be pointed out that the value of the interaction parameter changes with the reference size chosen for the elements of the lattice (see SI-A). A quantity that describes the miscibility of a specific material pair is rather the ratio between χ_12_ and the molar volume of the Flory–Huggins lattice sites *v*_0_. To explain this further, one may consider the free energy density Δ*G*_*V*_ which, being an intensive quantity, does not change with the total volume of the system. To express Δ*G*_*V*_, Δ*G* can first be adapted in order to reason in terms of number of moles,

4 $$\Delta G=\mathrm{RT}\left[n_{1}\ln\left(\phi_{1}\right)+n_{2}\ln\left(\phi_{2}\right)+n_{0}\phi_{1}\phi_{2}\chi_{12}\right]$$

with *n*_0_ the mole number of lattice sites and *n*_1_ and *n*_2_ the mole numbers of species 1 and 2, respectively. Δ*G*_*V*_ can then be obtained by dividing this latter equation by the volume of the mixture *V* = *n*_0_v_0_ and substituting *n*_1_ and *n*_2_ by making use of relations between the mole numbers, the volume fractions, and the sizes of both components in terms of number of occupied lattice sites *N*_1_ and *N*_2_, that is

5 $$n_{1}N_{1}=n_{0}\phi_{1},,n_{2}N_{2}=n_{0}\phi_{2}$$

The free energy density Δ*G*_*V*_ reads

6 $$\Delta G_{V}=\frac{\mathrm{RT}}{v_{0}}\left[\frac{\phi_{1}}{N_{1}}\ln\left(\phi_{1}\right)+\frac{\phi_{2}}{N_{2}}\ln\left(\phi_{2}\right)+\phi_{1}\phi_{2}\chi_{12}\right]$$

Considering now the mixing of a same material blend projected onto two different Flory–Huggins lattices (denoted hereafter by the superscripts (1) and (2)) with distinct lattice element sizes (i.e., reference molar volumes *v*_0_^(1)^ and *v*_0_^(2)^, respectively), the following equation immediately arises since the interaction energy contributions computed relatively to both reference systems (*RTϕ*_1_ϕ_2_χ_12_^(1)^/*v*_0_^(1)^ and *RTϕ*_1_ϕ_2_χ_12_^(2)^/*v*_0_^(2)^) are still required to be equal:

7 $$\frac{\chi_{12}^{\left(1\right)}}{v_{0}^{\left(1\right)}}=\frac{\chi_{12}^{\left(2\right)}}{v_{0}^{\left(2\right)}}\Leftrightarrow \chi_{12}^{\left(2\right)}=\frac{v_{0}^{\left(2\right)}}{v_{0}^{\left(1\right)}}\chi_{12}^{\left(1\right)}$$

This provides a scaling relation that can be used to adapt the interaction parameter value from one reference lattice to another. Values of χ_12_ should therefore always be reported with the considered lattice molar volume *v*_0_ in order to allow for reliable comparisons between different miscibility experiments.

Finally, formulas for the chemical potentials of both components can be obtained by taking the partial derivatives of the free energy (eq 4) with respect to the corresponding mole numbers *n*_1_ and *n*_2_. Note that the dependencies of the volume fractions ϕ_1_ and ϕ_2_ on *n*_1_ and *n*_2_ are taken into account during this calculation. Using eq 5 and the fact that ϕ_1_ + ϕ_2_ = 1, the expressions of the chemical potentials μ_1_ and μ_2_ ultimately simplify to

8 $$\{\begin{array}{l} \mu_{1}=\frac{\partial \Delta G}{\partial n_{1}}=RT\left[\ln\left(\phi_{1}\right)+\left(1-\phi_{1}\right)\left(1-\frac{N_{1}}{N_{2}}\right)+N_{1}\chi_{12}{\left(1-\phi_{1}\right)}^{2}\right]\\ \mu_{2}=\frac{\partial \Delta G}{\partial n_{2}}=RT\left[\ln\left(\phi_{2}\right)+\left(1-\phi_{2}\right)\left(1-\frac{N_{2}}{N_{1}}\right)+N_{2}\chi_{12}{\left(1-\phi_{2}\right)}^{2}\right] \end{array}$$

Again, as for eq 1, it can be observed that the original treatment by Flory^1^ results in slightly different chemical potentials due to the implicit scaling of the lattice elements with the smallest component of the mixture (see SI-A for more details).

## Generalization for Crystalline Multicomponent Mixtures

After having presented the features and equations of the classical Flory–Huggins theory that are fundamental for the present endeavor, the current section addresses the generalization of the model for material blends with any number of amorphous and/or crystalline components. Conceptually, the followed approach is analogous to the method detailed by Rubinstein and Colby^12^ for the enthalpy part of the interaction parameter. In the present development, it is applied directly to the overall free energy of the system, which includes all possible enthalpic and entropic contributions. As compared to the original treatment, this implies the following supplementary assumptions:

1. In addition to the enthalpy, the global entropy of the system (at equilibrium) can be decomposed into a sum of respective entropic contributions from each pair of neighboring lattice sites that constitute the blend.
2. The free energy gain associated with crystallization (which relates to the overall latent heat release upon this phase transition) can be modeled as resulting from attractive interactions between nearest neighbors on the Flory–Huggins lattice, similarly to the mean-field approach that yields the definition of the classical Flory–Huggins interaction parameter (eq 2). Density changes occurring as a material transitions from the amorphous to the ordered state are neglected, so that the size coefficients *N*_*i*_ of the different species on the Flory–Huggins lattice remain constant. Moreover, the coordination number *z* is approximated to be invariant upon crystallization as well, even though crystalline components realistically adopt a spatial crystal lattice conformation, which is distinct from the imaginary Flory–Huggins lattice.

Conventionally, the free energy change upon mixing Δ*G* is written as

9 $$\Delta G=G-G^{\left(0\right)}$$

Here, *G* denotes the free energy after the mixing and crystallization processes have occurred and *G* ^(0)^ is the reference free energy of the system in the unmixed amorphous state. Being extensive quantities, these total free energies can be calculated by adding up all individual free energy contributions from the different sites that form the Flory–Huggins lattice. For convenience, this can be expressed in factorized forms as

10 $$\{\begin{array}{l} G=\sum_{i=1}^{n}\phi_{i}\left[\left(1-\psi_{i}\right)G_{i}^{\left(a\right)}+\psi_{i}G_{i}^{\left(c\right)}\right]\\ G^{\left(0\right)}=\sum_{i=1}^{n}\phi_{i}G_{i}^{\left(0\right)} \end{array}$$

The summation is over the *n* components of the blend and the free energy contributions pertaining to lattice sites filled with a given species *i* are weighted by its corresponding total volume fraction ϕ_*i*_. In the formula for *G*, it is additionally distinguished whether the elements are in the crystalline or in the amorphous state (see superscript indices (*c*) and (*a*), respectively). A supplementary variable ψ_*i*_ is introduced here to represent the relative crystallinity of species *i*. In this way, the products ϕ_*i*_ψ_*i*_ and ϕ_*i*_(1 – ψ_*i*_) give the volume fractions of crystalline and amorphous material *i*, respectively. These terms multiply the free energies *G*_*i*_^(*c*)^ and *G*_*i*_^(*a*)^ that belong to crystalline and amorphous lattice sites, so that their proportions in the blend are respected in the free energy formula. In comparison to *G*, a unique free energy contribution per species (*G*_*i*_^(0)^) is necessary to compute *G* ^(0)^ since all the components are still amorphous in the reference state.

The lattice site contributions *G*_*i*_^(*a*)^, *G*_*i*_^(*c*)^, and *G*_*i*_^(0)^ can now be developed further following the assumption that they arise from nearest-neighbor interactions:

11 $$\{\begin{array}{l} G_{i}^{\left(a\right)}=\frac{n_{0}N_{A}z}{2}\sum_{j=1}^{n}\phi_{j}\left[\left(1-\psi_{j}\right)G_{\mathrm{ij}}^{\left(\mathrm{aa}\right)}+\psi_{j}G_{\mathrm{ij}}^{\left(\mathrm{ac}\right)}\right]\\ G_{i}^{\left(c\right)}=\frac{n_{0}N_{A}z}{2}\sum_{j=1}^{n}\phi_{j}\left[\left(1-\psi_{j}\right)G_{\mathrm{ij}}^{\left(\mathrm{ca}\right)}+\psi_{j}G_{\mathrm{ij}}^{\left(\mathrm{cc}\right)}\right]\\ G_{i}^{\left(0\right)}=\frac{n_{0}N_{A}z}{2}\sum_{j=1}^{n}\delta_{\mathrm{ij}}G_{\mathrm{ij}}^{\left(\mathrm{aa}\right)}=\frac{n_{0}N_{A}z}{2}G_{\mathrm{ii}}^{\left(\mathrm{aa}\right)} \end{array}$$

For any site neighbor to the one filled with species *i* (for which either *G*_*i*_^(*a*)^, *G*_*i*_^(*c*)^, or *G*_*i*_^(0)^ is written), and occupied by species *j* (which can be any of the *n* constituents, *i* included), four different types of interactions can occur depending on the state of both elements. The possible pair combinations are amorphous–amorphous, amorphous–crystalline, crystalline–amorphous, or crystalline–crystalline. For each one, a corresponding free energy contribution is considered: *G*_*ij*_^(*aa*)^, *G*_*ij*_^(*ac*)^, *G*_*ij*_^(*ca*)^, and *G*_*ij*_^(*cc*)^. The subscript indices (*i*, *j*) refer to the components involved in the interaction while the superscript indices (*a*, *c*) specify their respective state. The distinction between the amorphous–crystalline and crystalline–amorphous contributions (*G*_*ij*_^(*ac*)^ and *G*_*ij*_^(*ca*)^) matters, since these are, a priori, not necessarily symmetric. Employing the same mean-field treatment as in the classical Flory–Huggins theory,^12^ these terms are weighted by the probability of encountering the associated nearest-neighbor couple, knowing already that the lattice site occupied by component *i* is involved in the pair.

It is important to note that the developments are carried out, here, from a global perspective where the mixture (with its amorphous and crystalline domains) is viewed as a whole bulk, as no further information is known, a priori, about the precise locations and spatial arrangements adopted by the crystalline species inside the system. Analogously to the original Flory–Huggins model for isotropic amorphous mixing, the aforementioned probabilities are then given by the average volume fractions and crystallinities of the blend components. Hence, the probability to have species *j* neighboring species *i* is its overall volume fraction ϕ_*j*_. In addition, the probability for *j* to be amorphous is 1 – ψ_*j*_, and ψ_*j*_ to be crystalline. As in eq 10, the free energy contributions *G*_*ij*_^(*aa*)^ and *G*_*ij*_^(*ca*)^ are thus scaled with ϕ_*j*_(1 – ψ_*j*_), while *G*_*ij*_^(*ac*)^ and *G*_*ij*_^(*cc*)^ are multiplied by ϕ_*j*_ψ_*j*_.

The assumed bulk perspective neglects that crystalline species arrange in specific spatial configurations (i.e., the crystal lattices), which may possibly result in a restriction of the mixing. Especially, on the microstructural level, this means that the probability to find a given species (in a given state) next to another one is not readily given by its overall volume fraction (multiplied by its crystallinity), as is the case for a randomly disordered mixture. A prerequisite to properly express this probability as a more sophisticated function of the volume fractions and the crystallinities is the knowledge of the crystal lattice arrangements adopted by the mixed components, their tolerance with respect to defects, and their capacity to accommodate foreign species. Refinements of the model may be undertaken for mixtures where details about these properties are available. As they are material specific, this is, however, outside of the present scope.

In the premixing configuration described by *G*_*i*_^(0)^, the probability to find component *j* next to component *i* can be expressed by the Kronecker symbol δ_*ij*_ since the constituents are only in contact with themselves. Moreover, the system is fully amorphous in this case, so only the *G*_*ij*_^(*aa*)^ contribution remains. Equation 11 is finally obtained by summing over the number of components *n*, multiplying by the number of neighbors per lattice site *z* times the total number of Flory–Huggins lattice elements *n̅*_0_ = *n*_0_ *N*_*A*_ (where *N*_*A*_ denotes the Avogadro constant), and dividing by 2 to avoid counting twice each pairwise interaction.

Subsequently, eq 11 can be substituted into eq 10:

12 $$\{\begin{array}{l} G=\frac{n_{0}N_{A}z}{2}\sum_{i=1}^{n}\sum_{j=1}^{n}\phi_{i}\phi_{j}\left[\left(1-\psi_{i}\right)\left(1-\psi_{j}\right)G_{\mathrm{ij}}^{\left(\mathrm{aa}\right)}+\left(1-\psi_{i}\right)\psi_{j}G_{\mathrm{ij}}^{\left(\mathrm{ac}\right)}+\psi_{i}\left(1-\psi_{j}\right)G_{\mathrm{ij}}^{\left(\mathrm{ca}\right)}+\psi_{i}\psi_{j}G_{\mathrm{ij}}^{\left(\mathrm{cc}\right)}\right]\\ G^{\left(0\right)}=\frac{n_{0}N_{A}z}{2}\sum_{i=1}^{n}\phi_{i}G_{\mathrm{ii}}^{\left(\mathrm{aa}\right)} \end{array}$$

A major assumption of the Flory–Huggins theory is that the lattice is regular and invariant upon mixing,^1,12^ so that the coordination number *z* stays constant. Under this hypothesis, subtracting *G* ^(0)^ from *G* leads to the relationship

13 $$\Delta G=\frac{n_{0}N_{A}z}{2}\left[\sum_{i=1}^{n}\left(\phi_{i}^{2}\left[{\left(1-\psi_{i}\right)}^{2}G_{\mathrm{ii}}^{\left(\mathrm{aa}\right)}+2\psi_{i}\left(1-\psi_{i}\right)G_{\mathrm{ii}}^{\left(\mathrm{ac}\right)}+\psi_{i}^{2}G_{\mathrm{ii}}^{\left(\mathrm{cc}\right)}\right]-\phi_{i}G_{\mathrm{ii}}^{\left(\mathrm{aa}\right)}\right)+\sum_{i=1}^{n}\sum_{j\neq i}^{n}\phi_{i}\phi_{j}\left[\left(1-\psi_{i}\right)\left(1-\psi_{j}\right)G_{\mathrm{ij}}^{\left(\mathrm{aa}\right)}\;+\left(1-\psi_{i}\right)\psi_{j}G_{\mathrm{ij}}^{\left(\mathrm{ac}\right)}+\psi_{i}\left(1-\psi_{j}\right)G_{\mathrm{ij}}^{\left(\mathrm{ca}\right)}+\psi_{i}\psi_{j}G_{\mathrm{ij}}^{\left(\mathrm{cc}\right)}\right]\right]$$

In eq 13, contributions from neighbors of the same component are grouped separately to utilize the here existing symmetry between amorphous–crystalline and crystalline–amorphous interactions, that is *G*_*ii*_^(*ac*)^ = *G*_*ii*_^(*ca*)^. From there, the aim is to recover the ideal mixing and interaction terms from the classical Flory–Huggins theory in order to ensure the consistency with the original model. First, the free energy contributions of the upper sum that involve crystalline elements are rewritten according to their deviation from the amorphous–amorphous interaction, i.e., *G*_*ii*_^(*ac*)^ = *G*_*ii*_^(*aa*)^ + Δ*G*_*ii*_^(*ac*)^ and *G*_*ii*_^(*cc*)^ = *G*_*ii*_^(*aa*)^ + Δ*G*_*ii*_^(*cc*)^ (with Δ*G*_*ii*_^(*ac*)^ and Δ*G*_*ii*_^(*cc*)^ the respective correction terms for amorphous–crystalline and crystalline–crystalline contributions), so that

14 $$\Delta G=\frac{n_{0}N_{A}z}{2}\left[\sum_{i=1}^{n}\left(\phi_{i}^{2}\left[2\psi_{i}\left(1-\psi_{i}\right)\Delta G_{\mathrm{ii}}^{\left(\mathrm{ac}\right)}+\psi_{i}^{2}\Delta G_{\mathrm{ii}}^{\left(\mathrm{cc}\right)}\right]-\phi_{i}\left(1-\phi_{i}\right)G_{\mathrm{ii}}^{\left(\mathrm{aa}\right)}\right)\;+\sum_{i=1}^{n}\sum_{j\neq i}^{n}\phi_{i}\phi_{j}\left[\left(1-\psi_{i}\right)\left(1-\psi_{j}\right)G_{\mathrm{ij}}^{\left(\mathrm{aa}\right)}+\left(1-\psi_{i}\right)\psi_{j}G_{\mathrm{ij}}^{\left(\mathrm{ac}\right)}+\psi_{i}\left(1-\psi_{j}\right)G_{\mathrm{ij}}^{\left(\mathrm{ca}\right)}+\psi_{i}\psi_{j}G_{\mathrm{ij}}^{\left(\mathrm{cc}\right)}\right]\right]$$

Utilizing the fact that adding up all volume fractions always amounts to 1, and thus that 1 – ϕ_*i*_ = ∑_*j*≠*i*_^*n*^ϕ_*j*_, it is possible to transfer the term in *G*_*ii*_^(*aa*)^ into the double sum. There, it may also be distributed as follows among the different contributions since (1 – ψ_*i*_)­(1 – ψ_*j*_) + (1 – ψ_*i*_)­ψ_*j*_ + ψ_*i*_(1 – ψ_*j*_) + ψ_*i*_ψ_*j*_ = 1:

$$\Delta G=\frac{n_{0}N_{A}z}{2}\left[\overset{n}{\underset{i=1}{\sum }}\phi_{i}^{2}\left[2\psi_{i}\left(1-\psi_{i}\right)\Delta G_{\mathrm{ii}}^{\left(\mathrm{ac}\right)}+\psi_{i}^{2}\Delta G_{\mathrm{ii}}^{\left(\mathrm{cc}\right)}\right]+\overset{n}{\underset{i=1}{\sum }}\overset{n}{\underset{j\neq i}{\sum }}\phi_{i}\phi_{j}\left[\left(1-\psi_{i}\right)\left(1-\psi_{j}\right)\left(G_{\mathrm{ij}}^{\left(\mathrm{aa}\right)}-G_{\mathrm{ii}}^{\left(\mathrm{aa}\right)}\right)\;+\left(1-\psi_{i}\right)\psi_{j}\left(G_{\mathrm{ij}}^{\left(\mathrm{ac}\right)}-G_{\mathrm{ii}}^{\left(\mathrm{aa}\right)}\right)\;+\psi_{i}\left(1-\psi_{j}\right)\left(G_{\mathrm{ij}}^{\left(\mathrm{ca}\right)}-G_{\mathrm{ii}}^{\left(\mathrm{aa}\right)}\right)+\psi_{i}\psi_{j}\left(G_{\mathrm{ij}}^{\left(\mathrm{cc}\right)}-G_{\mathrm{ii}}^{\left(\mathrm{aa}\right)}\right)\right]\right]$$ 15

At this point, it is useful to express the free energy contributions of the second row in terms of their enthalpic (e.g., *H*_*ij*_^(*aa*)^) and entropic parts. The latter are additionally split into the entropy rise expected upon ideal mixing (*S*_*ij*_^(*id*)^) and a correction term (e.g., Δ*S*_*ij*_^(*aa*)^) accounting for potential deviations from this behavior, yielding

16 $$\{\begin{array}{l} G_{\mathrm{ij}}^{\left(\mathrm{aa}\right)}=H_{\mathrm{ij}}^{\left(\mathrm{aa}\right)}-T\left(S_{\mathrm{ij}}^{\left(\mathrm{id}\right)}+\Delta S_{\mathrm{ij}}^{\left(\mathrm{aa}\right)}\right)\\ G_{\mathrm{ij}}^{\left(\mathrm{ac}\right)}=H_{\mathrm{ij}}^{\left(\mathrm{ac}\right)}-T\left(S_{\mathrm{ij}}^{\left(\mathrm{id}\right)}+\Delta S_{\mathrm{ij}}^{\left(\mathrm{ac}\right)}\right)\\ G_{\mathrm{ij}}^{\left(\mathrm{ca}\right)}=H_{\mathrm{ij}}^{\left(\mathrm{ca}\right)}-T\left(S_{\mathrm{ij}}^{\left(\mathrm{id}\right)}+\Delta S_{\mathrm{ij}}^{\left(\mathrm{ca}\right)}\right)\\ G_{\mathrm{ij}}^{\left(\mathrm{cc}\right)}=H_{\mathrm{ij}}^{\left(\mathrm{cc}\right)}-T\left(S_{\mathrm{ij}}^{\left(\mathrm{id}\right)}+\Delta S_{\mathrm{ij}}^{\left(\mathrm{cc}\right)}\right) \end{array}$$

Replacing this into eq 15 and rearranging in order to regroup the ideal mixing entropies at the front of the double sum, one obtains

17 $$\Delta G=\frac{n_{0}N_{A}z}{2}\left[\sum_{i=1}^{n}\phi_{i}^{2}\left[2\psi_{i}\left(1-\psi_{i}\right)\Delta G_{\mathrm{ii}}^{\left(\mathrm{ac}\right)}+\psi_{i}^{2}\Delta G_{\mathrm{ii}}^{\left(\mathrm{cc}\right)}\right]+\sum_{i=1}^{n}\sum_{j\neq i}^{n}\phi_{i}\phi_{j}\left[-T\left(S_{\mathrm{ij}}^{\left(\mathrm{id}\right)}-S_{\mathrm{ii}}^{\left(\mathrm{id}\right)}\right)\;+\left(1-\psi_{i}\right)\left(1-\psi_{j}\right)\left(H_{\mathrm{ij}}^{\left(\mathrm{aa}\right)}-T\Delta S_{\mathrm{ij}}^{\left(\mathrm{aa}\right)}-H_{\mathrm{ii}}^{\left(\mathrm{aa}\right)}+T\Delta S_{\mathrm{ii}}^{\left(\mathrm{aa}\right)}\right)+\left(1-\psi_{i}\right)\psi_{j}\left(H_{\mathrm{ij}}^{\left(\mathrm{ac}\right)}-T\Delta S_{\mathrm{ij}}^{\left(\mathrm{ac}\right)}-H_{\mathrm{ii}}^{\left(\mathrm{aa}\right)}+T\Delta S_{\mathrm{ii}}^{\left(\mathrm{aa}\right)}\right)+\psi_{i}\left(1-\psi_{j}\right)\left(H_{\mathrm{ij}}^{\left(\mathrm{ca}\right)}-T\Delta S_{\mathrm{ij}}^{\left(\mathrm{ca}\right)}-H_{\mathrm{ii}}^{\left(\mathrm{aa}\right)}+T\Delta S_{\mathrm{ii}}^{\left(\mathrm{aa}\right)}\right)+\psi_{i}\psi_{j}\left(H_{\mathrm{ij}}^{\left(\mathrm{cc}\right)}-T\Delta S_{\mathrm{ij}}^{\left(\mathrm{cc}\right)}-H_{\mathrm{ii}}^{\left(\mathrm{aa}\right)}+T\Delta S_{\mathrm{ii}}^{\left(\mathrm{aa}\right)}\right)\right]\right]$$

In the case of an ideal amorphous mixture, the free energy reduces to the latter mentioned entropy contributions, which must therefore identify with logarithmic terms comparable to those presented in eq 4 to conform with the original model. Hence,

18 $$-\frac{Tn_{0}N_{A}z}{2}\sum_{i=1}^{n}\sum_{j\neq i}^{n}\phi_{i}\phi_{j}\left(S_{\mathrm{ij}}^{\left(\mathrm{id}\right)}-S_{\mathrm{ii}}^{\left(\mathrm{id}\right)}\right)=\mathrm{RT}\sum_{i=1}^{n}n_{i}\ln\left(\phi_{i}\right)$$

More details about the implied formulas for *S*_*ij*_^(*id*)^ and *S*_*ii*_^(*id*)^ can be found in the SI but are not necessary for the upcoming discussions (SI–B). Assuming all terms due to interactions between two different blend components *i* and *j* possess a symmetrical expression, i.e., *H*_*ij*_^(*aa*)^ – *T*Δ*S*_*ij*_^(*aa*)^ = *H*_*ji*_^(*aa*)^ – *T*Δ*S*_*ji*_^(*aa*)^, *H*_*ij*_^(*ac*)^ – *T*Δ*S*_*ij*_^(*ac*)^ = *H*_*ji*_^(*ca*)^ – *T*Δ*S*_*ji*_^(*ca*)^, *H*_*ij*_^(*ca*)^ – *T*Δ*S*_*ij*_^(*ca*)^ = *H*_*ji*_^(*ac*)^ – *T*Δ*S*_*ji*_^(*ac*)^, and *H*_*ij*_^(*cc*)^ – *T*Δ*S*_*ij*_^(*cc*)^ = *H*_*ji*_^(*cc*)^ – *T*Δ*S*_*ji*_^(*cc*)^, it can be observed that these actually appear twice in the remainder of the double sum. Thus, eq 17 can equivalently be rewritten as

$$\Delta G=\frac{n_{0}N_{A}z}{2}\overset{n}{\underset{i=1}{\sum }}\phi_{i}^{2}\left[2\psi_{i}\left(1-\psi_{i}\right)\Delta G_{\mathrm{ii}}^{\left(\mathrm{ac}\right)}+\psi_{i}^{2}\Delta G_{\mathrm{ii}}^{\left(\mathrm{cc}\right)}\right]+\mathrm{RT}\overset{n}{\underset{i=1}{\sum }}n_{i}\ln\left(\phi_{i}\right)+\frac{n_{0}N_{A}z}{2}\overset{n}{\underset{i=1}{\sum }}\overset{n}{\underset{j>i}{\sum }}\phi_{i}\phi_{j}[\left(1-\psi_{i}\right)\left(1-\psi_{j}\right)\left(2H_{\mathrm{ij}}^{\left(\mathrm{aa}\right)}-2T\Delta S_{\mathrm{ij}}^{\left(\mathrm{aa}\right)}-H_{\mathrm{ii}}^{\left(\mathrm{aa}\right)}-H_{\mathrm{jj}}^{\left(\mathrm{aa}\right)}+T\Delta S_{\mathrm{ii}}^{\left(\mathrm{aa}\right)}+T\Delta S_{\mathrm{jj}}^{\left(\mathrm{aa}\right)}\right)+\left(1-\psi_{i}\right)\psi_{j}\left(2H_{\mathrm{ij}}^{\left(\mathrm{ac}\right)}-2T\Delta S_{\mathrm{ij}}^{\left(\mathrm{ac}\right)}-H_{\mathrm{ii}}^{\left(\mathrm{aa}\right)}-H_{\mathrm{jj}}^{\left(\mathrm{aa}\right)}+T\Delta S_{\mathrm{ii}}^{\left(\mathrm{aa}\right)}+T\Delta S_{\mathrm{jj}}^{\left(\mathrm{aa}\right)}\right)+\psi_{i}\left(1-\psi_{j}\right)\left(2H_{\mathrm{ij}}^{\left(\mathrm{ca}\right)}-2T\Delta S_{\mathrm{ij}}^{\left(\mathrm{ca}\right)}-H_{\mathrm{ii}}^{\left(\mathrm{aa}\right)}-H_{\mathrm{jj}}^{\left(\mathrm{aa}\right)}+T\Delta S_{\mathrm{ii}}^{\left(\mathrm{aa}\right)}+T\Delta S_{\mathrm{jj}}^{\left(\mathrm{aa}\right)}\right)+\psi_{i}\psi_{j}\left(2H_{\mathrm{ij}}^{\left(\mathrm{cc}\right)}-2T\Delta S_{\mathrm{ij}}^{\left(\mathrm{cc}\right)}-H_{\mathrm{ii}}^{\left(\mathrm{aa}\right)}-H_{\mathrm{jj}}^{\left(\mathrm{aa}\right)}+T\Delta S_{\mathrm{ii}}^{\left(\mathrm{aa}\right)}+T\Delta S_{\mathrm{jj}}^{\left(\mathrm{aa}\right)}\right)]$$ 19

This allows to define interaction parameters for the four types of nearest-neighbor configurations that can occur (i.e., amorphous–amorphous, amorphous–crystalline, crystalline–amorphous, and crystalline–crystalline):

20 $$\{\begin{array}{l} \chi_{\mathrm{ij}}^{\left(\mathrm{aa}\right)}≔\frac{z}{\mathrm{kT}}[H_{\mathrm{ij}}^{\left(\mathrm{aa}\right)}-\frac{1}{2}\left(H_{\mathrm{ii}}^{\left(\mathrm{aa}\right)}+H_{\mathrm{jj}}^{\left(\mathrm{aa}\right)}\right)-T\left(\Delta S_{\mathrm{ij}}^{\left(\mathrm{aa}\right)}-\frac{1}{2}\left(\Delta S_{\mathrm{ii}}^{\left(\mathrm{aa}\right)}+\Delta S_{\mathrm{jj}}^{\left(\mathrm{aa}\right)}\right)\right)]\\ \chi_{\mathrm{ij}}^{\left(\mathrm{ac}\right)}≔\frac{z}{\mathrm{kT}}[H_{\mathrm{ij}}^{\left(\mathrm{ac}\right)}-\frac{1}{2}\left(H_{\mathrm{ii}}^{\left(\mathrm{aa}\right)}+H_{\mathrm{jj}}^{\left(\mathrm{aa}\right)}\right)-T\left(\Delta S_{\mathrm{ij}}^{\left(\mathrm{ac}\right)}-\frac{1}{2}\left(\Delta S_{\mathrm{ii}}^{\left(\mathrm{aa}\right)}+\Delta S_{\mathrm{jj}}^{\left(\mathrm{aa}\right)}\right)\right)]\\ \chi_{\mathrm{ij}}^{\left(\mathrm{ca}\right)}≔\frac{z}{\mathrm{kT}}[H_{\mathrm{ij}}^{\left(\mathrm{ca}\right)}-\frac{1}{2}\left(H_{\mathrm{ii}}^{\left(\mathrm{aa}\right)}+H_{\mathrm{jj}}^{\left(\mathrm{aa}\right)}\right)-T\left(\Delta S_{\mathrm{ij}}^{\left(\mathrm{ca}\right)}-\frac{1}{2}\left(\Delta S_{\mathrm{ii}}^{\left(\mathrm{aa}\right)}+\Delta S_{\mathrm{jj}}^{\left(\mathrm{aa}\right)}\right)\right)]\\ \chi_{\mathrm{ij}}^{\left(\mathrm{cc}\right)}≔\frac{z}{\mathrm{kT}}[H_{\mathrm{ij}}^{\left(\mathrm{cc}\right)}-\frac{1}{2}\left(H_{\mathrm{ii}}^{\left(\mathrm{aa}\right)}+H_{\mathrm{jj}}^{\left(\mathrm{aa}\right)}\right)-T\left(\Delta S_{\mathrm{ij}}^{\left(\mathrm{cc}\right)}-\frac{1}{2}\left(\Delta S_{\mathrm{ii}}^{\left(\mathrm{aa}\right)}+\Delta S_{\mathrm{jj}}^{\left(\mathrm{aa}\right)}\right)\right)] \end{array}$$

The first parameter, χ_*ij*_^(*aa*)^, is analogous to the interaction parameter from the classical Flory–Huggins theory and directly takes the expected linear form in 1/*T* (see eq 3). As do the supplementary parameters χ_*ij*_^(*ac*)^, χ_*ij*_^(*ca*)^, and χ_*ij*_^(*cc*)^, that stem from the extension for crystalline components. No explicit composition-dependencies arise from the present treatment, but it can be reminded that all the involved enthalpy and entropy terms may possibly be more complex functions of concentration, temperature, crystallinity, and material properties such as the polymer chain length, for example. Introducing the interaction parameters into eq 19 results in

21 $$\Delta G=\frac{n_{0}N_{A}z}{2}\sum_{i=1}^{n}\phi_{i}^{2}\left[2\psi_{i}\left(1-\psi_{i}\right)\Delta G_{\mathrm{ii}}^{\left(\mathrm{ac}\right)}+\psi_{i}^{2}\Delta G_{\mathrm{ii}}^{\left(\mathrm{cc}\right)}\right]+\mathrm{RT}\sum_{i=1}^{n}n_{i}\ln\left(\phi_{i}\right)+n_{0}\mathrm{RT}\sum_{i=1}^{n}\sum_{j>i}^{n}\phi_{i}\phi_{j}\left[\left(1-\psi_{i}\right)\left(1-\psi_{j}\right)\chi_{\mathrm{ij}}^{\left(\mathrm{aa}\right)}+\left(1-\psi_{i}\right)\psi_{j}\chi_{\mathrm{ij}}^{\left(\mathrm{ac}\right)}+\psi_{i}\left(1-\psi_{j}\right)\chi_{\mathrm{ij}}^{\left(\mathrm{ca}\right)}+\psi_{i}\psi_{j}\chi_{\mathrm{ij}}^{\left(\mathrm{cc}\right)}\right]$$

Alternatively, it is also possible to describe the amorphous–crystalline, crystalline–amorphous, and crystalline–crystalline interactions relatively to the amorphous–amorphous ones via corresponding corrective parameters, namely

22 $$\{\begin{array}{l} \Delta \chi_{\mathrm{ij}}^{\left(\mathrm{ac}\right)}≔\chi_{\mathrm{ij}}^{\left(\mathrm{ac}\right)}-\chi_{\mathrm{ij}}^{\left(\mathrm{aa}\right)}=\frac{z}{\mathrm{kT}}\left[\Delta H_{\mathrm{ij}}^{\left(\mathrm{ac}\right)}-T\left(\Delta S_{\mathrm{ij}}^{\left(\mathrm{ac}\right)}-\Delta S_{\mathrm{ij}}^{\left(\mathrm{aa}\right)}\right)\right]\\ \Delta \chi_{\mathrm{ij}}^{\left(\mathrm{ca}\right)}≔\chi_{\mathrm{ij}}^{\left(\mathrm{ca}\right)}-\chi_{\mathrm{ij}}^{\left(\mathrm{aa}\right)}=\frac{z}{\mathrm{kT}}\left[\Delta H_{\mathrm{ij}}^{\left(\mathrm{ca}\right)}-T\left(\Delta S_{\mathrm{ij}}^{\left(\mathrm{ca}\right)}-\Delta S_{\mathrm{ij}}^{\left(\mathrm{aa}\right)}\right)\right]\\ \Delta \chi_{\mathrm{ij}}^{\left(\mathrm{cc}\right)}≔\chi_{\mathrm{ij}}^{\left(\mathrm{cc}\right)}-\chi_{\mathrm{ij}}^{\left(\mathrm{aa}\right)}=\frac{z}{\mathrm{kT}}\left[\Delta H_{\mathrm{ij}}^{\left(\mathrm{cc}\right)}-T\left(\Delta S_{\mathrm{ij}}^{\left(\mathrm{cc}\right)}-\Delta S_{\mathrm{ij}}^{\left(\mathrm{aa}\right)}\right)\right] \end{array}$$

with Δ*H*_*ij*_^(*ac*)^ = *H*_*ij*_^(*ac*)^ – *H*_*ij*_^(*aa*)^, Δ*H*_*ij*_^(*ca*)^ = *H*_*ij*_^(*ca*)^ – *H*_*ij*_^(*aa*)^, and Δ*H*_*ij*_^(*cc*)^ = *H*_*ij*_^(*cc*)^ – *H*_*ij*_^(*aa*)^.

The total free energy upon mixing and crystallization then rather writes

23 $$\Delta G=\frac{n_{0}N_{A}z}{2}\sum_{i=1}^{n}\phi_{i}^{2}\left[2\psi_{i}\left(1-\psi_{i}\right)\Delta G_{\mathrm{ii}}^{\left(\mathrm{ac}\right)}+\psi_{i}^{2}\Delta G_{\mathrm{ii}}^{\left(\mathrm{cc}\right)}\right]\;+\mathrm{RT}\sum_{i=1}^{n}n_{i}\ln\left(\phi_{i}\right)+n_{0}\mathrm{RT}\sum_{i=1}^{n}\sum_{j>i}^{n}\phi_{i}\phi_{j}\left[\chi_{\mathrm{ij}}^{\left(\mathrm{aa}\right)}\;+\left(1-\psi_{i}\right)\psi_{j}\Delta \chi_{\mathrm{ij}}^{\left(\mathrm{ac}\right)}+\psi_{i}\left(1-\psi_{j}\right)\Delta \chi_{\mathrm{ij}}^{\left(\mathrm{ca}\right)}+\psi_{i}\psi_{j}\Delta \chi_{\mathrm{ij}}^{\left(\mathrm{cc}\right)}\right]$$

Note that, in either of both forms, all interaction parameters are still subjected to the scaling with the reference size of the Flory–Huggins lattice elements (see discussion around eq 7 and SI-A).

Since the derivation of Δ*G* follows from a perspective where the mixture is considered as a whole bulk, it can be remarked that the newly introduced interaction parameters are defined irrespective of the presence of phase interfaces within the system (analogously to the classical Flory–Huggins interaction parameter, which does not require an amorphous–amorphous phase separation to exist). This is important, as the model is sought to be general enough to capture a wide variety of blend behaviors, so that it must be able to not only represent pure crystals, but also impure ones with defects, and mixed cocrystals, where several chemical species undergo crystallization together and share the same crystal lattice.

Thus, it can be emphasized that a free energy with nonzero χ_*ij*_^(*ac*)^, χ_*ij*_^(*ca*)^, and χ_*ij*_^(*cc*)^ (or Δ*χ*_*ij*_^(*ac*)^, Δ*χ*_*ij*_^(*ca*)^, and Δ*χ*_*ij*_^(*cc*)^) interaction parameters is not necessarily synonymous for multiphase systems. For example, crystal–amorphous interaction parameters contribute to the description of the free energy of mixtures where a given material can form a crystal lattice through covalent bonding, and other species are able to fit on interstitial sites without disrupting the lattice structure (thereby, leading only to a single macroscopic phase). Likewise, in blends in which the species are compatible for cocrystallization, the crystal–crystal interaction parameter affects the total free energy, without implying the formation of distinct crystalline phases separated by interfaces.

All terms from the original theory are now recovered in eqs 21 and 23. The last steps of this derivation focus on incorporating as well the molar latent heats of the crystallizing species Δ*h*_*i*_, which are commonly employed in models of the crystallization phase transition.^36,39^ In a fully crystallized one-component system, Δ*G*_*ii*_^(*cc*)^ can for instance be related to Δ*h*_*i*_ by equating either eqs 21 or 23 (reduced with *n* = 1, ψ_*i*_ = 1, and ϕ_*i*_ = 1, which implies here that *n*_0_ = *n*_*i*_ *N*_*i*_, *N*_*i*_ standing for the size of species *i* in terms of Flory–Huggins lattice elements) with the usual linear approximation of the crystallization free energy made in the vicinity of the equilibrium melting temperature *T*_*m*,*i*_:^38,44^

24 $$\frac{n_{0}N_{A}z}{2}\Delta G_{\mathrm{ii}}^{\left(\mathrm{cc}\right)}=n_{i}\Delta h_{i}\left(1-\frac{T}{T_{m,i}}\right)\;\Leftrightarrow \Delta G_{\mathrm{ii}}^{\left(\mathrm{cc}\right)}=\frac{2\Delta h_{i}}{N_{i}N_{A}z}\left(1-\frac{T}{T_{m,i}}\right)$$

Moreover, one can also introduce a molar energy parameter Δ*σ*_*i*_ similar to Δ*h*_*i*_, so as to define Δ*G*_*ii*_^(*ac*)^ as

25 $$\Delta G_{\mathrm{ii}}^{\left(\mathrm{ac}\right)}≔\frac{\Delta \sigma_{i}}{N_{i}N_{A}z}$$

Comparably to the classical Flory–Huggins theory, the present model describes the free energy within the bulk of a multicomponent system. This means that free energy contributions arising at phase interfaces are not fully captured here. Therefore, theoretical frameworks used to simulate the dynamics of multiphase systems require additional terms, as for instance highlighted in the derivation of the Cahn–Hilliard equation that governs the time evolution of the local composition in nonhomogeneous mixtures.^45^ Nevertheless, it can be pointed out that the Δ*σ*_*i*_ parameter controls the strength of the interaction between crystalline and amorphous elements of the same species, and is, thus, also decisive for the interfacial energy existing between a crystal and its surrounding amorphous phase. In accordance with expectations from crystallization modeling experiments,^36,46,47^ it is assumed to bear a dependency on the system temperature. Even though its definition differs from that of the other interaction parameters, Δ*σ*_*i*_ has in fact a comparable nature. In effect, χ_*ij*_^(*aa*)^ can also be shown to be responsible for interface energy properties that arise between two amorphous phases in a demixing-prone blend.^48,49^ In the same way, χ_*ij*_^(*ac*)^, χ_*ij*_^(*ca*)^, and χ_*ij*_^(*cc*)^ (or Δ*χ*_*ij*_^(*ac*)^, Δ*χ*_*ij*_^(*ca*)^, and Δ*χ*_*ij*_^(*cc*)^) are anticipated to be determinant for the surface energy at amorphous–crystalline, crystalline–amorphous, and crystalline–crystalline phase interfaces.

Under the hypothesis that Δ*G*_*ii*_^(*cc*)^ and Δ*G*_*ii*_^(*ac*)^ are constants at fixed temperature, eqs 24 and 25 can readily be introduced in the free energy expressions (eqs 21 and 23), giving

$$\Delta G=n_{0}\sum_{i=1}^{n}\frac{\phi_{i}^{2}}{N_{i}}\left[\psi_{i}\left(1-\psi_{i}\right)\Delta \sigma_{i}+\psi_{i}^{2}\Delta h_{i}\left(1-\frac{T}{T_{m,i}}\right)\right]+RT\sum_{i=1}^{n}n_{i}\ln\left(\phi_{i}\right)+n_{0}RT\sum_{i=1}^{n}\sum_{j>i}^{n}\phi_{i}\phi_{j}\left[\left(1-\psi_{i}\right)\left(1-\psi_{j}\right)\chi_{\mathrm{ij}}^{\left(\mathrm{aa}\right)}+\left(1-\psi_{i}\right)\psi_{j}\chi_{\mathrm{ij}}^{\left(\mathrm{ac}\right)}+\psi_{i}\left(1-\psi_{j}\right)\chi_{\mathrm{ij}}^{\left(\mathrm{ca}\right)}+\psi_{i}\psi_{j}\chi_{\mathrm{ij}}^{\left(\mathrm{cc}\right)}\right]$$ 26

and

27 $$\Delta G=n_{0}\sum_{i=1}^{n}\frac{\phi_{i}^{2}}{N_{i}}\left[\psi_{i}\left(1-\psi_{i}\right)\Delta \sigma_{i}+\psi_{i}^{2}\Delta h_{i}\left(1-\frac{T}{T_{m,i}}\right)\right]+RT\sum_{i=1}^{n}n_{i}\ln\left(\phi_{i}\right)+n_{0}RT\sum_{i=1}^{n}\sum_{j>i}^{n}\phi_{i}\phi_{j}\left[\chi_{\mathrm{ij}}^{\left(\mathrm{aa}\right)}+\left(1-\psi_{i}\right)\psi_{j}\Delta \chi_{\mathrm{ij}}^{\left(\mathrm{ac}\right)}+\psi_{i}\left(1-\psi_{j}\right)\Delta \chi_{\mathrm{ij}}^{\left(\mathrm{ca}\right)}+\psi_{i}\psi_{j}\Delta \chi_{\mathrm{ij}}^{\left(\mathrm{cc}\right)}\right]$$

In the broadest case, it can nevertheless be expected that Δ*G*_*ii*_^(*cc*)^ and Δ*G*_*ii*_^(*ac*)^ exhibit more complex dependencies on blend composition, crystallinity, and material properties. More sophisticated crystallization models are then required to identify these terms with measurable quantities. Recalling that, in general, *n*_0_ϕ_*i*_ = *n*_*i*_ *N*_*i*_ (see eq 5), a final adjustment of the crystallization free energy is performed in eqs 26 and 26a, which yields

28 $$\Delta G=\sum_{i=1}^{n}n_{i}\phi_{i}\left[\psi_{i}\left(1-\psi_{i}\right)\Delta \sigma_{i}+\psi_{i}^{2}\Delta h_{i}\left(1-\frac{T}{T_{m,i}}\right)\right]+RT\sum_{i=1}^{n}n_{i}\ln\left(\phi_{i}\right)+n_{0}RT\sum_{i=1}^{n}\sum_{j>i}^{n}\phi_{i}\phi_{j}\left[\left(1-\psi_{i}\right)\left(1-\psi_{j}\right)\chi_{\mathrm{ij}}^{\left(\mathrm{aa}\right)}+\left(1-\psi_{i}\right)\psi_{j}\chi_{\mathrm{ij}}^{\left(\mathrm{ac}\right)}+\psi_{i}\left(1-\psi_{j}\right)\chi_{\mathrm{ij}}^{\left(\mathrm{ca}\right)}+\psi_{i}\psi_{j}\chi_{\mathrm{ij}}^{\left(\mathrm{cc}\right)}\right]$$

and

29 $$\Delta G=\sum_{i=1}^{n}n_{i}\phi_{i}\left[\psi_{i}\left(1-\psi_{i}\right)\Delta \sigma_{i}+\psi_{i}^{2}\Delta h_{i}\left(1-\frac{T}{T_{m,i}}\right)\right]+RT\sum_{i=1}^{n}n_{i}\ln\left(\phi_{i}\right)+n_{0}RT\sum_{i=1}^{n}\sum_{j>i}^{n}\phi_{i}\phi_{j}\left[\chi_{\mathrm{ij}}^{\left(\mathrm{aa}\right)}+\left(1-\psi_{i}\right)\psi_{j}\Delta \chi_{\mathrm{ij}}^{\left(\mathrm{ac}\right)}+\psi_{i}\left(1-\psi_{j}\right)\Delta \chi_{\mathrm{ij}}^{\left(\mathrm{ca}\right)}+\psi_{i}\psi_{j}\Delta \chi_{\mathrm{ij}}^{\left(\mathrm{cc}\right)}\right]$$

## Chemical Potential and Phase Diagram Calculations

The extended free energy also allows for chemical potentials μ_*i*_ = ∂Δ*G*/∂*n*_*i*_ to be calculated for each of the *n* blend components, namely

$$\mu_{i}=\phi_{i}\left(2-\phi_{i}\right)\left[\psi_{i}\left(1-\psi_{i}\right)\Delta \sigma_{i}+\psi_{i}^{2}\Delta h_{i}\left(1-\frac{T}{T_{m,i}}\right)\right]-\overset{n}{\underset{j\neq i}{\sum }}\left(\phi_{j}^{2}\frac{N_{i}}{N_{j}}\left[\psi_{j}\left(1-\psi_{j}\right)\Delta \sigma_{j}+\psi_{j}^{2}\Delta h_{j}\left(1-\frac{T}{T_{m,j}}\right)\right]\right)+\mathrm{RT}\left[\ln\left(\phi_{i}\right)+\overset{n}{\underset{j\neq i}{\sum }}\phi_{j}\left(1-\frac{N_{i}}{N_{j}}\right)+N_{i}\overset{n}{\underset{j\neq i}{\sum }}\phi_{j}\left(\overset{n}{\underset{k\neq i}{\sum }}\phi_{k}\left[\left(1-\psi_{i}\right)\left(1-\psi_{k}\right)\chi_{\mathrm{ik}}^{\left(\mathrm{aa}\right)}+\left(1-\psi_{i}\right)\psi_{k}\chi_{\mathrm{ik}}^{\left(\mathrm{ac}\right)}+\psi_{i}\left(1-\psi_{k}\right)\chi_{\mathrm{ik}}^{\left(\mathrm{ca}\right)}+\psi_{i}\psi_{k}\chi_{\mathrm{ik}}^{\left(\mathrm{cc}\right)}\right]-\overset{n}{\underset{\begin{array}{l} k>j\\ k\neq i \end{array}}{\sum }}\phi_{k}\left[\left(1-\psi_{j}\right)\left(1-\psi_{k}\right)\chi_{\mathrm{jk}}^{\left(\mathrm{aa}\right)}+\left(1-\psi_{j}\right)\psi_{k}\chi_{\mathrm{jk}}^{\left(\mathrm{ac}\right)}+\psi_{j}\left(1-\psi_{k}\right)\chi_{\mathrm{jk}}^{\left(\mathrm{ca}\right)}+\psi_{j}\psi_{k}\chi_{\mathrm{jk}}^{\left(\mathrm{cc}\right)}\right]\right)\right]$$ 30

or

31 $$\mu_{i}=\phi_{i}\left(2-\phi_{i}\right)\left[\psi_{i}\left(1-\psi_{i}\right)\Delta \sigma_{i}+\psi_{i}^{2}\Delta h_{i}\left(1-\frac{T}{T_{m,i}}\right)\right]-\sum_{j\neq i}^{n}\left(\phi_{j}^{2}\frac{N_{i}}{N_{j}}\left[\psi_{j}\left(1-\psi_{j}\right)\Delta \sigma_{j}+\psi_{j}^{2}\Delta h_{j}\left(1-\frac{T}{T_{m,j}}\right)\right]\right)+\mathrm{RT}\left[\ln\left(\phi_{i}\right)+\sum_{j\neq i}^{n}\phi_{j}\left(1-\frac{N_{i}}{N_{j}}\right)+N_{i}\sum_{j\neq i}^{n}\phi_{j}\left(\sum_{k\neq i}^{n}\phi_{k}\left[\chi_{\mathrm{ik}}^{\left(\mathrm{aa}\right)}+\left(1-\psi_{i}\right)\psi_{k}\Delta \chi_{\mathrm{ik}}^{\left(\mathrm{ac}\right)}+\psi_{i}\left(1-\psi_{k}\right)\Delta \chi_{\mathrm{ik}}^{\left(\mathrm{ca}\right)}+\psi_{i}\psi_{k}\Delta \chi_{\mathrm{ik}}^{\left(\mathrm{cc}\right)}\right]-\sum_{\begin{array}{l} k>j\\ k\neq i \end{array}}^{n}\phi_{k}\left[\chi_{\mathrm{jk}}^{\left(\mathrm{aa}\right)}+\left(1-\psi_{j}\right)\psi_{k}\Delta \chi_{\mathrm{jk}}^{\left(\mathrm{ac}\right)}+\psi_{j}\left(1-\psi_{k}\right)\Delta \chi_{\mathrm{jk}}^{\left(\mathrm{ca}\right)}+\psi_{j}\psi_{k}\Delta \chi_{\mathrm{jk}}^{\left(\mathrm{cc}\right)}\right]\right)\right]$$

depending on whether the first (eq 27) or the second (eq 27a) form is considered for Δ*G* (see SI–C for the detailed chemical potential derivation). In the case of an amorphous binary system (where *n* = 2, ψ_1_ = ψ_2_ = 0, and ϕ_2_ = 1 – ϕ_1_), it can be verified that these expressions reduce to eq 8, as expected.

Furthermore, for a two-component mixture in which solely one of the species is subject to crystallization and eventually forms a pure crystalline phase at equilibrium with a remaining mixed amorphous phase, it is possible to recover the melting point depression formula^1^ that can be utilized to assess the value of the classical Flory–Huggins interaction parameter from experimental liquidus measurements. For this, the chemical potential of the crystallizing species is evaluated with eq 28 (or eq 29) separately in both phases. In what follows, it is assumed without loss of generality that species 1 is the crystallizing one. Moreover, quantities pertaining to the crystalline and the amorphous phase are referenced by the superscripts (*c*) and (*a*), respectively. Equating both chemical potentials (thus denoted by μ_1_^(*c*)^ and μ_1_^(*a*)^), one obtains

$$\mu_{1}^{\left(c\right)}=\mu_{1}^{\left(a\right)}\Leftrightarrow \frac{\Delta h_{1}}{R}(\frac{1}{T}-\frac{1}{T_{m,1}})=\ln\left(\phi_{1}^{\left(a\right)}\right)+\left(1-\phi_{1}^{\left(a\right)}\right)\left(1-\frac{N_{1}}{N_{2}}\right)+N_{1}\chi_{12}{\left(1-\phi_{1}^{\left(a\right)}\right)}^{2}$$ 32

Note that the assumption that the crystalline phase is pure and perfectly ordered is crucial here because it implies that ϕ_1_^(*c*)^ = 1 and ψ_1_^(*c*)^ = 1, so that ultimately μ_1_^(*c*)^ = Δ*h*_1_ (1 – *T*/*T*_*m*,1_). Otherwise, the equation of the chemical potentials μ_1_^(*c*)^ and μ_1_^(*a*)^ becomes more complex and effects related to the crystalline–amorphous interaction parameter also come into play. Upon additional simplifications that differentiate between polymers and small molecules, this general formula (eq 30) can further be transformed into the commonly employed relationships presented by Nishi and Wang.^50^

Besides predicting the melting point depression, the present free energy model can also be used to produce phase diagrams. To do so, it is convenient to rely on the free energy density:

33 $$\Delta G_{V}=\sum_{i=1}^{n}\frac{\phi_{i}^{2}}{v_{i}}\left[\psi_{i}\left(1-\psi_{i}\right)\Delta \sigma_{i}+\psi_{i}^{2}\Delta h_{i}\left(1-\frac{T}{T_{m,i}}\right)\right]+\frac{\mathrm{RT}}{v_{0}}\sum_{i=1}^{n}\frac{\phi_{i}}{N_{i}}\ln\left(\phi_{i}\right)+\frac{\mathrm{RT}}{v_{0}}\sum_{i=1}^{n}\sum_{j>i}^{n}\phi_{i}\phi_{j}\left[\left(1-\psi_{i}\right)\left(1-\psi_{j}\right)\chi_{\mathrm{ij}}^{\left(\mathrm{aa}\right)}+\left(1-\psi_{i}\right)\psi_{j}\chi_{\mathrm{ij}}^{\left(\mathrm{ac}\right)}+\psi_{i}\left(1-\psi_{j}\right)\chi_{\mathrm{ij}}^{\left(\mathrm{ca}\right)}+\psi_{i}\psi_{j}\chi_{\mathrm{ij}}^{\left(\mathrm{cc}\right)}\right]$$

or

34 $$\Delta G_{V}=\sum_{i=1}^{n}\frac{\phi_{i}^{2}}{v_{i}}\left[\psi_{i}\left(1-\psi_{i}\right)\Delta \sigma_{i}+\psi_{i}^{2}\Delta h_{i}\left(1-\frac{T}{T_{m,i}}\right)\right]+\frac{\mathrm{RT}}{v_{0}}\sum_{i=1}^{n}\frac{\phi_{i}}{N_{i}}\ln\left(\phi_{i}\right)+\frac{\mathrm{RT}}{v_{0}}\sum_{i=1}^{n}\sum_{j>i}^{n}\phi_{i}\phi_{j}\left[\chi_{\mathrm{ij}}^{\left(\mathrm{aa}\right)}+\left(1-\psi_{i}\right)\psi_{j}\Delta \chi_{\mathrm{ij}}^{\left(\mathrm{ac}\right)}+\psi_{i}\left(1-\psi_{j}\right)\Delta \chi_{\mathrm{ij}}^{\left(\mathrm{ca}\right)}+\psi_{i}\psi_{j}\Delta \chi_{\mathrm{ij}}^{\left(\mathrm{cc}\right)}\right]$$

where *v*_*i*_ = *v*_0_ *N*_*i*_ stands for the molar volume of species *i*.

In this work, the convex hull approach^9,51–53^ is used to determine the different regions of the phase diagrams, although other methods exist as well.^54,55^ All the diagrams presented hereafter are calculated from the second free energy form (eq 31a). Nonetheless, exactly the same figures can be achieved with the alternative form (eq 31) and the adequate interaction parameters χ_*ij*_^(*ac*)^, χ_*ij*_^(*ca*)^, and χ_*ij*_^(*cc*)^, instead of Δ*χ*_*ij*_^(*ac*)^, Δ*χ*_*ij*_^(*ca*)^, and Δ*χ*_*ij*_^(*cc*)^, respectively. The reason to rather use the second form over the first is that additional interactions involving crystalline components are considered relatively to the strength of the amorphous–amorphous ones, which facilitates the exploration of the parameter space. With the first formula, parameter combinations that cause atypical diagram shapes, and are not expected for most physical systems, are more likely to be encountered. For example, when employing moderate values of χ_*ij*_^(*aa*)^ associated with relatively low χ_*ij*_^(*ca*)^, χ_*ij*_^(*ac*)^, and χ_*ij*_^(*cc*)^, the free energy may favor a crystalline state at equilibrium, even without any crystallization driving force (i.e., at vanishing Δ*h*_*i*_(1 – *T*/*T*_*m*,*i*_)). To obtain this with eq 31a, one would need to explicitly counter the magnitude of χ_*ij*_^(*aa*)^ with accordingly negative correction parameters Δ*χ*_*ij*_^(*ac*)^, Δ*χ*_*ij*_^(*ca*)^, and Δ*χ*_*ij*_^(*cc*)^.

Figures 2, 3, and 4 display typical examples of diagram shapes for binary systems. In Figure 2, mixtures prone to amorphous demixing without any crystallization phase transition are modeled. The location of the spinodal and binodal gaps depends exclusively on the values of χ_12_^(*aa*)^, *N*_1_, and *N*_2_, as already established within the framework of the classical Flory–Huggins theory.^12,56^ Upper and lower critical solution temperature behavior (UCST and LCST) is obtained when using either a positive or a negative *B* coefficient in the formula for χ_12_^(*aa*)^ (eq 3). In addition, in the LCST case, *A* has to be higher than the critical χ_12_^(*aa*)^ value^12,37^ above which the blend is susceptible to demix.

**Figure 2:**
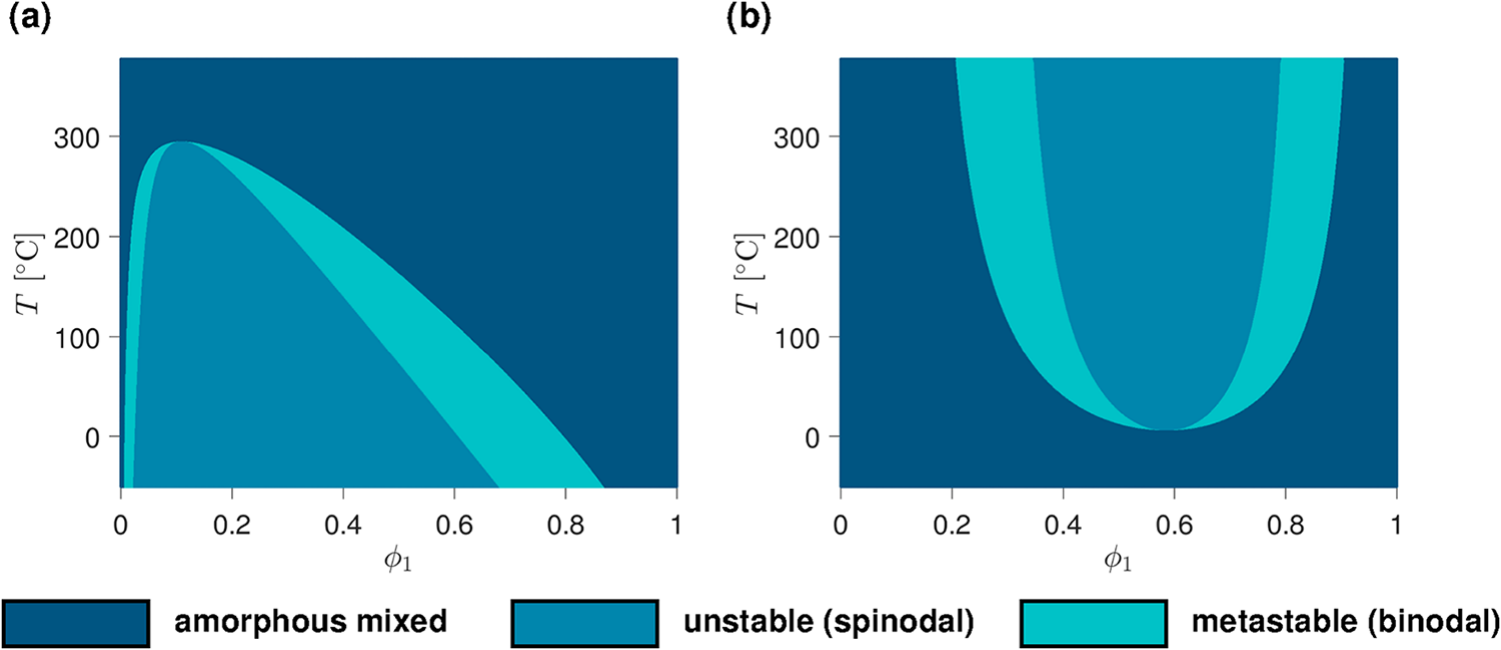
Phase diagrams of binary mixtures subject to (a) UCST-type and (b) LCST-type amorphous demixing. In (a), the blend is strongly asymmetric (*N*_1_ = 100 and *N*_2_ = 1), which results in a miscibility gap that leans toward compositions richer in the smaller constituent. In (b), the asymmetry is not as severe (*N*_1_ = 1 and *N*_2_ = 2) and the immiscible region is accordingly more centered. All relevant parameters used for the calculation of the diagrams are provided in the SI (SI-D).

**Figure 3:**
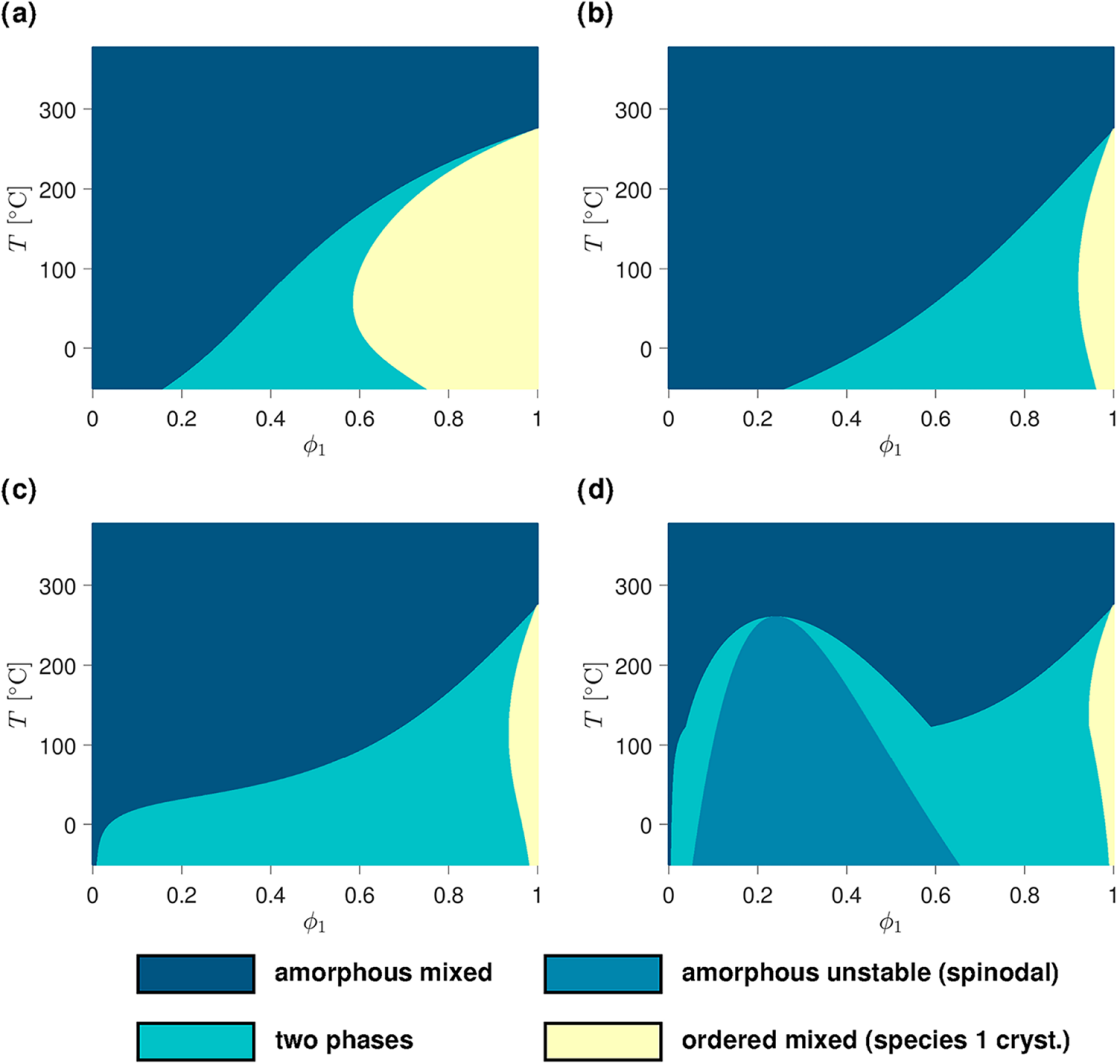
Phase diagrams of binary mixtures containing one species that can crystallize. In the first row, only the crystalline–amorphous parameter Δ*χ*_12_^(*ca*)^ is nonzero and varied according to (a) Δ*χ*_12_^(*ca*)^ = 100/*T* and (b) Δ*χ*_12_^(*ca*)^ = 0.2 + 350/*T*. In the second row, Δ*χ*_12_^(*ca*)^ is maintained at 0.2 + 350/*T* while the effect of amorphous–amorphous interactions ranging from (c) χ_12_^(*aa*)^ = 0.3 + 110/*T* to (d) χ_12_^(*aa*)^ = 0.4 + 250/*T* is added. All relevant parameters used for the calculation of the diagrams are provided in the SI (SI-D).

**Figure 4:**
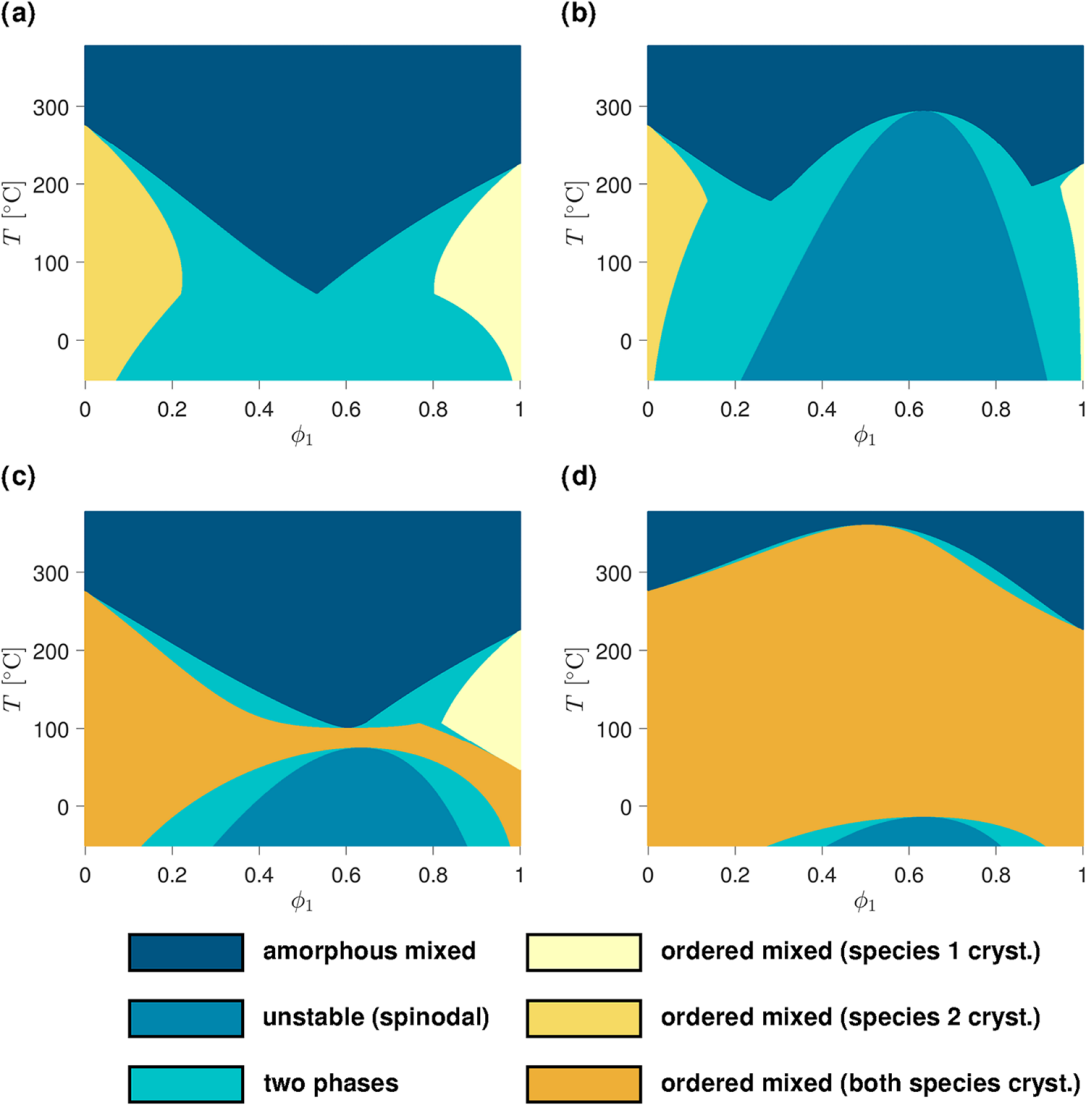
Phase diagrams of binary mixtures where both components can crystallize. In the first row, the amorphous–amorphous, amorphous–crystalline, and crystalline–amorphous interaction parameters are nonzero (i.e., χ_12_^(*aa*)^, Δ*χ*_12_^(*ac*)^, and Δ*χ*_12_^(*ca*)^). χ_12_^(*aa*)^ is varied according to (a) χ_12_^(*aa*)^ = 0.3 + 150/*T* and (b) χ_12_^(*aa*)^ = 0.4 + 480/*T*, while Δ*χ*_12_^(*ac*)^ and Δ*χ*_12_^(*ca*)^ are maintained at Δ*χ*_12_^(*ac*)^ = 0.1 + 100/*T* and Δ*χ*_12_^(*ca*)^ = 0.05 + 50/*T*, respectively. In the second row, the effect of crystalline–crystalline compatibility is added. Δ*χ*_12_^(*cc*)^ is accordingly set to (c) Δ*χ*_12_^(*cc*)^ = 0.3 – 30/*T* and to (d) Δ*χ*_12_^(*cc*)^ = – 60/*T*. In both (c) and (d), the values of χ_12_^(*aa*)^, Δ*χ*_12_^(*ac*)^, and Δ*χ*_12_^(*ca*)^ are the same as in (a). All relevant parameters used for the calculation of the diagrams are provided in the SI (SI-D).

Figure 3 shows diagrams for blends containing one crystallizing species. The effect of Δ*χ*_12_^(*ca*)^ is isolated in Figure 3a and b. It can be seen that increasing the values of the entropy and enthalpy contributions of the crystalline–amorphous interaction parameter shifts the two-phase domain toward higher concentrations of the crystalline component. Additionally, the gap widens and the liquidus becomes concave at most volume fractions (except close to ϕ = 0 and possibly ϕ = 1). For most crystallizing mixtures, it is however anticipated that both χ_12_^(*aa*)^ and Δ*χ*_12_^(*ca*)^ are nonzero. Adding a χ_12_^(*aa*)^ with a positive *B* coefficient expands the two-phase region (Figure 3c) and can cause an amorphous immiscibility region to emerge above the liquidus (Figure 3d).

Figure 4 now addresses the situation where both components can crystallize. Figure 4a demonstrates a typical diagram shape with a eutectic point obtained for moderate χ_12_^(*aa*)^, Δ*χ*_12_^(*ac*)^, and Δ*χ*_12_^(*ca*)^ values. Figure 4b then illustrates the possible interplay with an amorphous demixing region induced by a relatively high χ_12_^(*aa*)^. In both Figure 4a and b, no crystalline–crystalline interactions are considered, and the ordered phases arise from the crystallization of only one of the two components. The other species may be mixed into this ordered phase (for instance as defects or on interstitial sites of the crystal lattice) but does not explicitly form bonds and generate a latent heat release.

Starting from the parameter set of Figure 4a, c, and d exemplify how the phase equilibria evolve when Δ*χ*_12_^(*cc*)^ becomes progressively more negative, that is the blended materials become increasingly more compatible in the crystalline state. It can be seen that this triggers the appearance of a region where both components contribute together to the crystallization process, thus forming cocrystals. Moreover, the slopes of the melting point depressions are damped (Figure 4c) and ultimately inverted, leading to higher melting temperatures in the blend as compared to the pure materials (Figure 4d). It can also be remarked that these diagrams predict a miscibility gap where spinodal/binodal phase separation takes place in the ordered state.

Comparing the diagram types produced from this model with the prior one of Matkar and Kyu,^32^ it can be seen that both lead to similar features. A notable distinction is that diagrams computed from the model of Matkar and Kyu tend to exhibit fully ordered phases below a certain threshold temperature, even at vanishing content of the actual crystallizing components, as depicted in the SI (SI-E). This feature is not anticipated for most physical systems and is also not witnessed with the current free energy.

Another qualitative difference concerns the mathematical form of the crystallization energy (see SI-E). With the present formulation, it varies with the square of the crystallinity. In contrast, the framework of Matkar and Kyu relies on a Landau expansion^34^ which employs polynomials of higher order. It is shown in the SI how this latter approach can also be incorporated into the formulas developed here (SI-E), resulting in the following free energy densities:

35 $$\Delta G_{V}=\sum_{i=1}^{n}\frac{\phi_{i}^{2}}{v_{i}}\left[\psi_{i}^{2}{\left(1-\psi_{i}\right)}^{2}\Delta \sigma_{i}+\psi_{i}^{2}\left(3-2\psi_{i}\right)\Delta h_{i}\left(1-\frac{T}{T_{m,i}}\right)\right]+\frac{\mathrm{RT}}{v_{0}}\sum_{i=1}^{n}\frac{\phi_{i}}{N_{i}}\ln\left(\phi_{i}\right)+\frac{\mathrm{RT}}{v_{0}}\sum_{i=1}^{n}\sum_{j>i}^{n}\phi_{i}\phi_{j}\left[\left(1-\psi_{i}\right)\left(1-\psi_{j}\right)\chi_{\mathrm{ij}}^{\left(\mathrm{aa}\right)}+\left(1-\psi_{i}\right)\psi_{j}\chi_{\mathrm{ij}}^{\left(\mathrm{ac}\right)}+\psi_{i}\left(1-\psi_{j}\right)\chi_{\mathrm{ij}}^{\left(\mathrm{ca}\right)}+\psi_{i}\psi_{j}\chi_{\mathrm{ij}}^{\left(\mathrm{cc}\right)}\right]$$

and

36 $$\Delta G_{V}=\sum_{i=1}^{n}\frac{\phi_{i}^{2}}{v_{i}}\left[\psi_{i}^{2}{\left(1-\psi_{i}\right)}^{2}\Delta \sigma_{i}+\psi_{i}^{2}\left(3-2\psi_{i}\right)\Delta h_{i}\left(1-\frac{T}{T_{m,i}}\right)\right]+\frac{\mathrm{RT}}{v_{0}}\sum_{i=1}^{n}\frac{\phi_{i}}{N_{i}}\ln\left(\phi_{i}\right)+\frac{\mathrm{RT}}{v_{0}}\sum_{i=1}^{n}\sum_{j>i}^{n}\phi_{i}\phi_{j}\left[\chi_{\mathrm{ij}}^{\left(\mathrm{aa}\right)}+\left(1-\psi_{i}\right)\psi_{j}\Delta \chi_{\mathrm{ij}}^{\left(\mathrm{ac}\right)}+\psi_{i}\left(1-\psi_{j}\right)\Delta \chi_{\mathrm{ij}}^{\left(\mathrm{ca}\right)}+\psi_{i}\psi_{j}\Delta \chi_{\mathrm{ij}}^{\left(\mathrm{cc}\right)}\right]$$

Phase diagrams generated from these expressions possess qualitative properties comparable to those already presented and are therefore not discussed. Nevertheless, the free energy forms stemming from the Landau theory predict a so-called “spinodal temperature”^36^ below which the crystallization energy barrier vanishes, so that the phase transition may proceed spontaneously without following a nucleation and growth process. This is not the case with the current free energy density (eqs 31 and 31a), where the barrier always exists until *T* = 0 K (see discussion in SI-E).

Finally, phase diagrams computed for ternary systems are visualized in Figure 5. Figure 5a depicts an amorphous mixture with a range of ternary compositions that are prone to phase separation despite all binary material combinations being fully miscible. Figure 5b illustrates how amorphous miscibility gaps can overlap, leading either to binary or ternary phase equilibria with associated regions for binary and ternary spinodal decomposition.

**Figure 5:**
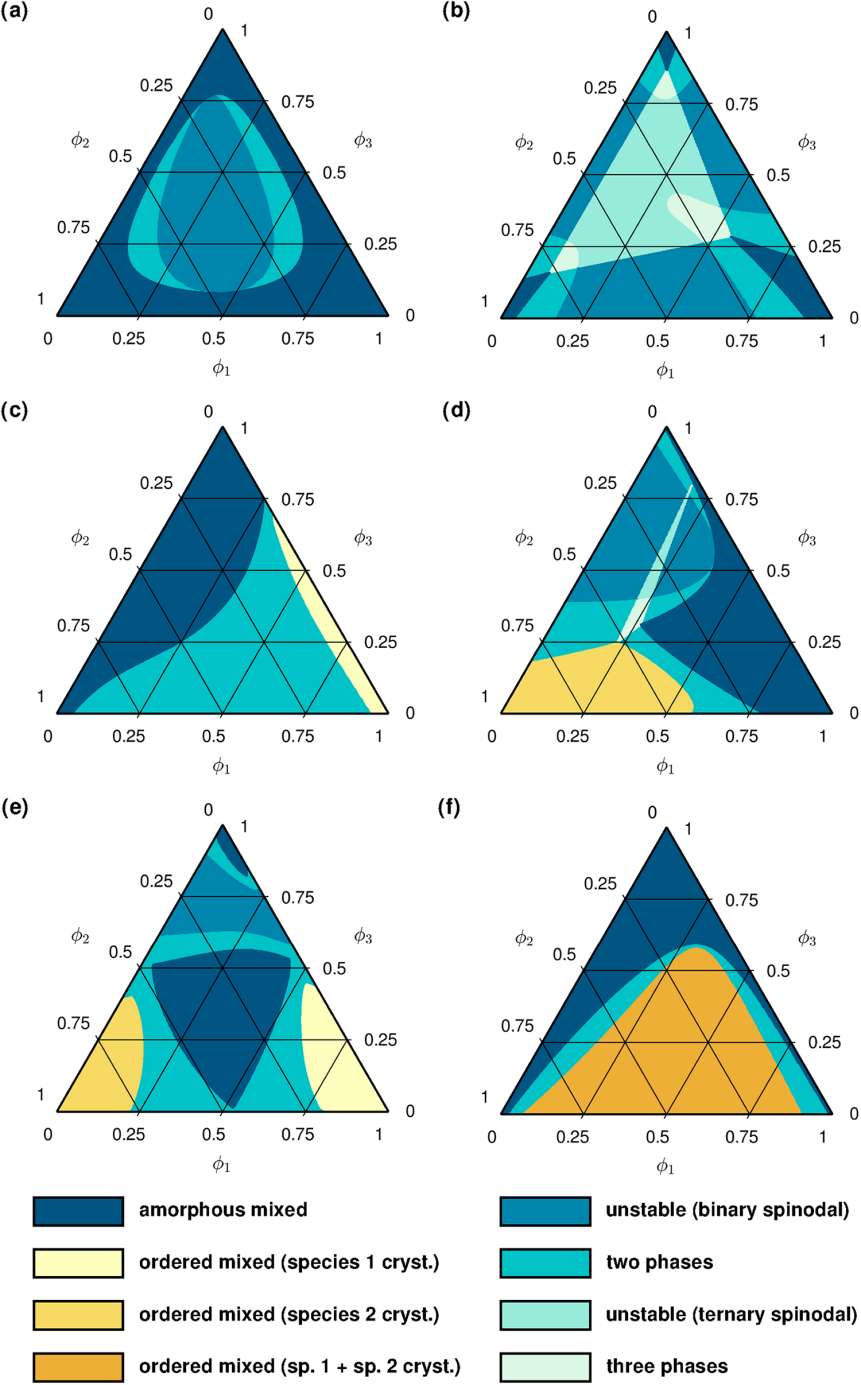
Phase diagrams of ternary mixtures exhibiting various distinct types of phase equilibria. All relevant parameters used for the calculation of the diagrams are provided in the SI (SI-D).

Figure 5c and d then display selected crystallization configurations that involve only one crystalline component. In Figure 5c, the crystallizing species tolerates a higher impurity content of the third material on its crystal lattice, as compared to the second one. As a result, the two-phase region widens when the overall composition is close to that of a binary blend of components 1 and 2, and narrows progressively as it approaches the axis where the second species vanishes. In Figure 5d, the amorphous–amorphous interaction parameter between the second and the third constituent is sufficiently high to trigger the appearance of an amorphous demixing region. In this particular case, the interplay of all the different interaction parameters causes a domain with a ternary phase equilibrium consisting of one crystalline and two amorphous phases.

The mixtures represented in Figure 5e and f include two species subject to crystallization. The three blend constituents in Figure 5e are moderately incompatible both in the amorphous and/or in the crystalline state, so that all two-phase regions bridge from one diagram boundary to another. The central part of the diagram predicts a mixed amorphous phase due to the amorphous–amorphous interaction parameters being still low enough and the amorphous–crystalline and crystalline–amorphous ones being sufficiently high. Its area, however, tends to reduce when the former increase or the latter decrease. In comparison, the last figure (Figure 5f) presents a situation where the crystallizing components demonstrate relatively high compatibility in the ordered state. As already seen in Figure 4d for a binary blend, this can permit a composition range with a stable crystalline phase even above the melting temperatures of both pure materials.

All in all, these results demonstrate that a large variety of systems can be modeled with the derived free energy formulas. It may be mentioned that this showcase presentation of binary and ternary phase diagrams is by no means exhaustive and that many more shapes are available. Moreover, it has also to be stressed that all employed interaction parameters follow the linear form in 1/*T* with constant coefficients (eq 3). Allowing these to be more complex functions of temperature, composition and/or material properties is expected to extend the range of accessible blend behaviors even further.

## Conclusion

To summarize, this work presented a general free energy model describing the thermodynamics of mixing of crystalline multicomponent blends. By extending the mean-field approach commonly employed to calculate enthalpic mixing interactions between two amorphous species, the well-established Flory–Huggins theory was augmented to account for mixtures that involve any number of constituents, all of which being allowed to undergo a crystallization phase transition. Expressions for the chemical potentials of the blend components were also obtained from the derived free energy. In the limit of binary mixtures that exhibit perfectly pure crystalline phases, the chemical potentials were verified to consistently recover the melting point depression formula from the original theoretical framework.

Notable features of the present model are the amorphous–crystalline, crystalline–amorphous, and crystalline–crystalline interaction parameters, which, in addition to the classical amorphous–amorphous one, determine miscibility properties between the mixed components depending on their respective state. A binary and ternary phase diagram showcase study demonstrated how these parameters impact phase separation phenomena that occur within blends. Depending on the interaction parameter values, interplay between miscibility gaps and melting point depressions, spinodal decomposition in the amorphous as well as in the crystalline state, and cocrystalline phase equilibria, can for instance be modeled.

An advantage of the current free energy formulation is to retain a relative simplicity, while being able to qualitatively represent various distinct and complex blend behaviors. It remains to be verified how accurately it can provide quantitative analyses for practical systems. Performing critical comparisons of model predictions against dedicated experimental measurements is therefore recommended for future investigations. For this, further formal examinations are also of interest to relate the expressions of the aforementioned interaction parameters to physical crystal characteristics, such as, for example, the global geometrical structure of the crystal lattices, the ability of their interstitial lattice sites to accommodate foreign blend species, or the predisposition of the mixed materials to form cocrystals.

## Supplementary Material



## Notes

Further Comments about the Classical Flory-Huggins Theory; Detailed Expression of the Ideal Mixing Entropy Terms; Derivation of the Generalized Chemical Potential Formulae; Parameters for Phase Diagram Calculations; Analysis of Free Energy Models basing on Landau Theory (PDF)
