# Supplementary material for: Extending the Flory–Huggins Theory for Crystalline Multicomponent Mixtures
Source: Macromolecules. 2026 Feb 11;59(4):1953–66. doi: 10.1021/acs.macromol.5c02297 (PMC12947687; doi:10.1021/acs.macromol.5c02297)
Supplement: Supplementary file 1 [file ma5c02297_si_001.pdf]

Extending the Flory-Huggins Theory for Crystalline Multicomponent Mixtures

Supplementary Information

Maxime Siber,*[a,b] Olivier J. J. Ronsin,[a] and Jens Harting [a,b,c]

[a] *Helmholtz Institute Erlangen-Nürnberg for Renewable Energy, Forschungszentrum Jülich, Fürther Straße 248, 90429 Nürnberg, Germany, E-mail: m.siber@fz-juelich.de*
[b] *Department of Chemical and Biological Engineering, Friedrich-Alexander-Universität Erlangen-Nürnberg, Fürther Straße 248, 90429 Nürnberg, Germany*
[c] *Department of Physics, Friedrich-Alexander-Universität Erlangen-Nürnberg, Fürther Straße 248, 90429 Nürnberg, Germany*

Contents

A Further Comments about the Classical Flory-Huggins Theory 1

B Detailed Expression of the Ideal Mixing Entropy Terms 2

C Derivation of the Generalized Chemical Potential Formulae 3

D Parameters for Phase Diagram Calculations 5

E Analysis of Free Energy Models basing on Landau Theory 7

References 10

A Further Comments about the Classical Flory-Huggins Theory

In several literature sources, including the reference book of Flory [1], the mixing free energy is introduced with a slightly different formula than the one presented here in the main manuscript, namely

$$\Delta G = kT\left[\overline{n}_1 \ln(\phi_1) + \overline{n}_2 \ln(\phi_2) + \overline{n}_1 \phi_2 \tilde{\chi}_{12}\right] \ . \tag{1}$$

This is because the derivation that leads to Eq. 1 is carried out from the perspective of species 1, which is assumed to be the smallest blend constituent and is therefore used to scale the elements of the Flory-Huggins lattice. Here, $\tilde{\chi}_{12}$ denotes the interaction parameter that arises in this development. However, one may equally write the free energy from the perspective of the second component, which then results in a switch of the indices in the last term and a different value for the interaction parameter. Utilizing that, more generally, $\overline{n}_0\phi_1 = \overline{n}_1 N_1$, Eq. 1 can rather be transformed into

$$\Delta G = kT\left[\overline{n}_1 \ln(\phi_1) + \overline{n}_2 \ln(\phi_2) + \overline{n}_0 \phi_1 \phi_2 \chi_{12}\right] \ , \tag{2}$$

with $\chi_{12} = \tilde{\chi}_{12}/N_1$. This expression is more convenient to highlight that the free energy is actually symmetric with respect to both components and that the value of the interaction parameter varies with the size chosen for the lattice elements. Recalling that the Flory-Huggins lattice is a virtual construct, it can be recognized that ΔG should be independent of its properties. In particular, this implies that the product $\overline{n}_0\chi_{12}$ is invariant for a given material pair when the blend volume is maintained constant. This holds irrespective of the specific value of $\overline{n}_0$, which is dependent on the size of the lattice elements and which can, in principle, be selected arbitrarily. χ_{12} has thus to vary inversely with $\overline{n}_0$ (as long as the total volume remains the same). The scaling relation of χ_{12} with the volume of the lattice elements can then be deduced from the free energy density, as shown in the main text.
Furthermore, calculating the chemical potentials directly from Eq. 1 yields the following relationships:

$$\begin{cases} \mu_1 = \dfrac{\partial \Delta G}{\partial n_1} = RT\left[\ln(\phi_1) + (1-\phi_1)\left(1 - \dfrac{N_1}{N_2}\right) + \tilde{\chi}_{12}(1-\phi_1)^2\right] \ , \\ \mu_2 = \dfrac{\partial \Delta G}{\partial n_2} = RT\left[\ln(\phi_2) + (1-\phi_2)\left(1 - \dfrac{N_2}{N_1}\right) + \dfrac{N_2}{N_1}\tilde{\chi}_{12}(1-\phi_2)^2\right] \ . \end{cases} \tag{3}$$

These may also be turned into the formulae featured in the main body of the article by substituting the interaction parameter $\tilde{\chi}_{12}$ with $N_1\chi_{12}$.

B Detailed Expression of the Ideal Mixing Entropy Terms

In ideal conditions, or when neglecting additional configurational and/or orientational effects, the number of microstates accessible before mixing for an element of species i on the Flory-Huggins lattice is equal to the number of sites times the overall volume fraction of i (i.e. $\overline{n}_0\phi_i$) [2]. Its associated entropy is thus given by $k\ln(\overline{n}_0\phi_i)$. Multiplying by the number of i elements $\overline{n}_i$ and summing over the total number of components n, the global entropy $S^{(0,id)}$ for the pre-mixed system writes

$$S^{(0,id)} = k\sum_{i=1}^{n}\overline{n}_i\ln\left(\overline{n}_0\phi_i\right) . \tag{4}$$

This entropy may as well be expressed according to the weighted sum approach followed in this work:

$$S^{(0,id)} = \frac{n_0N_Az}{2}\sum_{i=1}^{n}\phi_iS_{ii}^{(id)} , \tag{5}$$

where $S_{ii}^{(id)}$ is the ideal entropy contribution arising due to interactions between neighbours sites occupied by the same component. Since mixing has not yet taken place, both parts that involve species i in the sums of Eq. 4 and Eq. 5 must be equal, so that $S_{ii}^{(id)}$ can be identified as

$$S_{ii}^{(id)} = \frac{2k}{zN_i}\ln\left(\overline{n}_0\phi_i\right) . \tag{6}$$

Note that $\overline{n}_0 = n_0N_A$ and $\overline{n}_0\phi_i = \overline{n}_iN_i$ are utilized to reduce the equation. The $S_{ii}^{(id)}$ term is also present in the expression of the entropy once ideal mixing occurred and is involved in the following relationship that ensures the consistency with the classical Flory-Huggins theory, as discussed in the main article:

$$-\frac{n_0N_Az}{2}\sum_{i=1}^{n}\sum_{j\neq i}^{n}\phi_i\phi_j\left(S_{ij}^{(id)} - S_{ii}^{(id)}\right) = R\sum_{i=1}^{n}n_i\ln\left(\phi_i\right) . \tag{7}$$

Here, it is sought to detail as well $S_{ij}^{(id)}$, which, in contrast to $S_{ii}^{(id)}$, accounts for the entropy related to interactions between distinct components. First, Eq. 7 can be rearranged in order to isolate the $S_{ij}^{(id)}$ part on the left hand side, that is

$$\sum_{i=1}^{n}\sum_{j\neq i}^{n}\phi_i\phi_jS_{ij}^{(id)} = \sum_{i=1}^{n}\phi_i\left(\sum_{j\neq i}^{n}\phi_j\right)S_{ii}^{(id)} - \frac{2k}{n_0z}\sum_{i=1}^{n}n_i\ln\left(\phi_i\right) , \tag{8}$$

with $R = kN_A$. Next, assuming that $S_{ij}^{(id)}$ is symmetric (i.e. $S_{ij}^{(id)} = S_{ji}^{(id)}$), substituting the expression for $S_{ii}^{(id)}$ (Eq. 6), and recalling that $\sum_{j\neq i}^{n}\phi_j = (1-\phi_i)$, Eq. 8 can be rewritten as

$$\sum_{i=1}^{n}\sum_{j>i}^{n}\phi_i\phi_j2S_{ij}^{(id)} = \frac{2k}{z}\left[\sum_{i=1}^{n}\frac{\phi_i(1-\phi_i)\ln\left(\overline{n}_0\phi_i\right)}{N_i} - \sum_{i=1}^{n}\frac{n_i\ln\left(\phi_i\right)}{n_0}\right] . \tag{9}$$

By expanding the double sum of the left hand side, it can be seen that terms bearing the index of a given constituent appear exactly $n-1$ times. Under the hypothesis that ideal entropy contributions from interactions between different components all share the same mathematical description, regardless of the specific material combination, a summation term that involves species i and j can be expressed as

$$\phi_i\phi_jS_{ij}^{(id)} = \frac{k}{(n-1)z}\left[\frac{\phi_i(1-\phi_i)\ln\left(\overline{n}_0\phi_i\right)}{N_i} + \frac{\phi_j(1-\phi_j)\ln\left(\overline{n}_0\phi_j\right)}{N_j} - \frac{n_i\ln\left(\phi_i\right)}{n_0} - \frac{n_j\ln\left(\phi_j\right)}{n_0}\right] , \forall j\neq i . \tag{10}$$

Finally, dividing by the product $\phi_i\phi_j$, using $n_0\phi_i = n_iN_i$, and regrouping matching logarithmic terms, this equation can be simplified further to find a formula for $S_{ij}^{(id)}$:

$$S_{ij}^{(id)} = \frac{k}{(n-1)z}\left[\frac{\ln\left(\overline{n}_0\right) - \phi_i\ln\left(\overline{n}_0\phi_i\right)}{\phi_jN_i} + \frac{\ln\left(\overline{n}_0\right) - \phi_j\ln\left(\overline{n}_0\phi_j\right)}{\phi_iN_j}\right] , \forall j\neq i . \tag{11}$$

C Derivation of the Generalized Chemical Potential Formulae

The starting point of this derivation is the free energy formula for crystalline multicomponent mixtures featured in the main body of the article. The development is carried out here only for the first form of the free energy, as it is essentially the same for the second one which uniquely differs by the expression of the interaction parameters. For the sake of readability, the free energy ΔG is decomposed into three terms ($\mathcal{A}$, $\mathcal{B}$, and $\mathcal{C}$) that are treated separately in what follows:

$$\begin{aligned}\Delta G = & \underbrace{\sum_{i=1}^{n} n_i \phi_i \left[\psi_i(1-\psi_i)\Delta\sigma_i + \psi_i^2 \Delta h_i \left(1 - \frac{T}{T_{m,i}}\right)\right]}_{\mathcal{A}} + \underbrace{RT\sum_{i=1}^{n} n_i \ln(\phi_i)}_{\mathcal{B}} + \\ & \underbrace{n_0 RT \sum_{i=1}^{n}\sum_{j>i}^{n} \phi_i\phi_j \left[(1-\psi_i)(1-\psi_j)\chi_{ij}^{(aa)} + (1-\psi_i)\psi_j\chi_{ij}^{(ac)} + \psi_i(1-\psi_j)\chi_{ij}^{(ca)} + \psi_i\psi_j\chi_{ij}^{(cc)}\right]}_{\mathcal{C}} .\end{aligned} \tag{12}$$

The chemical potential of species i (denoted by μ_i) is then obtained by taking the partial derivative of ΔG with respect to the corresponding mole number n_i:

$$\mu_i = \frac{\partial \Delta G}{\partial n_i} = \frac{\partial \mathcal{A}}{\partial n_i} + \frac{\partial \mathcal{B}}{\partial n_i} + \frac{\partial \mathcal{C}}{\partial n_i} . \tag{13}$$

It can be seen that Eq. 12 includes volume fraction variables that are also varying with the mole numbers. Therefore, their derivatives are evaluated and substituted when encountered in the upcoming equations. Depending whether the considered volume fraction pertains to the component for which the chemical potential is calculated, or any other, the final form of its derivative diverges slightly, namely

$$\begin{cases} \dfrac{\partial \phi_i}{\partial n_i} = \dfrac{v_i}{\sum_{k=1}^{n} n_k v_k} - \dfrac{n_i v_i^2}{\left(\sum_{k=1}^{n} n_k v_k\right)^2} = \dfrac{(1-\phi_i)v_i}{\sum_{k=1}^{n} n_k v_k} = \dfrac{(1-\phi_i)\phi_i}{n_i} , \\ \dfrac{\partial \phi_j}{\partial n_i} = -\dfrac{n_j v_j v_i}{\left(\sum_{k=1}^{n} n_k v_k\right)^2} = -\dfrac{\phi_j v_i}{\sum_{k=1}^{n} n_k v_k} = -\dfrac{\phi_j \phi_i}{n_i} , \ \forall j \neq i. \end{cases} \tag{14}$$

The derivation of the part of the free energy that describes the crystallization phase transitions ($\mathcal{A}$) is now undertaken:

$$\begin{aligned}\frac{\partial \mathcal{A}}{\partial n_i} &= \frac{\partial (n_i\phi_i)}{\partial n_i}\left[\psi_i(1-\psi_i)\Delta\sigma_i + \psi_i^2\Delta h_i\left(1-\frac{T}{T_{m,i}}\right)\right] + \sum_{j\neq i}^{n} n_j \frac{\partial \phi_j}{\partial n_i}\left[\psi_j(1-\psi_j)\Delta\sigma_j + \psi_j^2\Delta h_j\left(1-\frac{T}{T_{m,j}}\right)\right] \\ &= \phi_i\left[\psi_i(1-\psi_i)\Delta\sigma_i + \psi_i^2\Delta h_i\left(1-\frac{T}{T_{m,i}}\right)\right] + \phi_i(1-\phi_i)\left[\psi_i(1-\psi_i)\Delta\sigma_i + \psi_i^2\Delta h_i\left(1-\frac{T}{T_{m,i}}\right)\right] \\ &\quad + \sum_{j\neq i}^{n} n_j \left(\frac{-\phi_j v_i}{\sum_{k=1}^{n} n_k v_k}\right)\left[\psi_j(1-\psi_j)\Delta\sigma_j + \psi_j^2\Delta h_j\left(1-\frac{T}{T_{m,j}}\right)\right] .\end{aligned} \tag{15}$$

Note that, in this work, all the parameters in the crystallization contributions, that is mainly the barrier $\Delta\sigma_j$ and the latent heat Δh_j (for $1 \leq j \leq n$), are assumed to be independent of the composition. Further simplifications can be made by pointing out that

$$\frac{n_j v_i}{\sum_{k=1}^{n} n_k v_k} = \phi_j \frac{v_i}{v_j} = \phi_j \frac{N_i}{N_j} , \tag{16}$$

which leads to

$$\frac{\partial \mathcal{A}}{\partial n_i} = \phi_i(2-\phi_i)\left[\psi_i(1-\psi_i)\Delta\sigma_i + \psi_i^2\Delta h_i\left(1-\frac{T}{T_{m,i}}\right)\right] - \sum_{j\neq 1}^{n} \phi_j^2 \frac{N_i}{N_j}\left[\psi_j(1-\psi_j)\Delta\sigma_j + \psi_j^2\Delta h_j\left(1-\frac{T}{T_{m,j}}\right)\right] . \tag{17}$$

The free energy term related to the ideal mixing entropy ($\mathcal{B}$) is subsequently derived:

$$
\begin{aligned}
\frac{\partial \mathcal{B}}{\partial n_i} &= RT\left[\frac{\partial(n_i \ln(\phi_i))}{\partial n_i} + \sum_{j\neq i}^{n} n_j \frac{\partial(\ln(\phi_j))}{\partial n_i}\right] \\
&= RT\left[\ln(\phi_i) + (1-\phi_i) - \sum_{j\neq i}^{n} \frac{n_j v_i}{\sum_{k=1}^{n} n_k v_k}\right] \ .
\end{aligned} \tag{18}
$$

Making use again of Eq. 16, along with $(1-\phi_i) = \sum_{j\neq i}^{n} \phi_j$ to regroup the last two terms, this reduces to

$$
\frac{\partial \mathcal{B}}{\partial n_i} = RT\left[\ln(\phi_i) + \sum_{j\neq i}^{n} \phi_j \left(1 - \frac{N_i}{N_j}\right)\right] \ . \tag{19}
$$

Before calculating the derivative of the free energy part relative to nearest-neighbour interactions ($\partial\mathcal{C}/\partial n_i$), $\mathcal{C}$ can be expanded and rearranged in order to extract specifically the terms involving the volume fraction of species i:

$$
\begin{aligned}
\mathcal{C} &= RTn_0 \sum_{j=1}^{n}\sum_{k>j}^{n} \phi_j\phi_k \left[(1-\psi_j)(1-\psi_k)\chi_{jk}^{(aa)} + (1-\psi_j)\psi_k\chi_{jk}^{(ac)} + \psi_j(1-\psi_k)\chi_{jk}^{(ca)} + \psi_j\psi_k\chi_{jk}^{(cc)}\right] \\
&= RTn_0 \left[\sum_{j=1}^{i-1}\left(\sum_{\substack{k>j\\k\neq i}}^{n} \phi_j\phi_k \left[(1-\psi_j)(1-\psi_k)\chi_{jk}^{(aa)} + (1-\psi_j)\psi_k\chi_{jk}^{(ac)} + \psi_j(1-\psi_k)\chi_{jk}^{(ca)} + \psi_j\psi_k\chi_{jk}^{(cc)}\right]\right.\right. \\
&\quad \left. + \ \phi_j\phi_i \left[(1-\psi_j)(1-\psi_i)\chi_{ji}^{(aa)} + (1-\psi_j)\psi_i\chi_{ji}^{(ac)} + \psi_j(1-\psi_i)\chi_{ji}^{(ca)} + \psi_j\psi_i\chi_{ji}^{(cc)}\right]\right) \\
&\quad + \sum_{j=i+1}^{n}\sum_{\substack{k>j\\k\neq i}}^{n} \phi_j\phi_k \left[(1-\psi_j)(1-\psi_k)\chi_{jk}^{(aa)} + (1-\psi_j)\psi_k\chi_{jk}^{(ac)} + \psi_j(1-\psi_k)\chi_{jk}^{(ca)} + \psi_j\psi_k\chi_{jk}^{(cc)}\right] \\
&\quad \left. + \sum_{k>i}^{n} \phi_i\phi_k \left[(1-\psi_i)(1-\psi_k)\chi_{ik}^{(aa)} + (1-\psi_i)\psi_k\chi_{ik}^{(ac)} + \psi_i(1-\psi_k)\chi_{ik}^{(ca)} + \psi_i\psi_k\chi_{ik}^{(cc)}\right]\right] \\
&= RTn_0 \left[\sum_{j\neq i}^{n} \phi_i\phi_j \left[(1-\psi_i)(1-\psi_j)\chi_{ij}^{(aa)} + (1-\psi_i)\psi_j\chi_{ij}^{(ac)} + \psi_i(1-\psi_j)\chi_{ij}^{(ca)} + \psi_i\psi_j\chi_{ij}^{(cc)}\right]\right. \\
&\quad \left. + \sum_{j\neq i}^{n}\sum_{\substack{k>j\\k\neq i}}^{n} \phi_j\phi_k \left[(1-\psi_j)(1-\psi_k)\chi_{jk}^{(aa)} + (1-\psi_j)\psi_k\chi_{jk}^{(ac)} + \psi_j(1-\psi_k)\chi_{jk}^{(ca)} + \psi_j\psi_k\chi_{jk}^{(cc)}\right]\right] \ .
\end{aligned} \tag{20}
$$

This is done in anticipation of the usage of Eq. 14, which distinguishes between ϕ_i and ϕ_j ($\forall\ j \neq i$). It is practical as well to replace directly $n_0\phi_j = n_j N_j$ at this point, so that the variation of n_0, implied by a change in n_i, does not have to be taken into account. The derivative then reads

$$
\begin{aligned}
\frac{\partial \mathcal{C}}{\partial n_i} = RT \Bigg[& \sum_{j\neq i}^{n} N_i \frac{\partial (n_i\phi_j)}{\partial n_i} \left[(1-\psi_i)(1-\psi_j)\chi_{ij}^{(aa)} + (1-\psi_i)\psi_j\chi_{ij}^{(ac)} + \psi_i(1-\psi_j)\chi_{ij}^{(ca)} + \psi_i\psi_j\chi_{ij}^{(cc)}\right] \\
& + \sum_{j\neq i}^{n} \sum_{\substack{k>j \\ k\neq i}}^{n} N_j n_j \frac{\partial \phi_k}{\partial n_i} \left[(1-\psi_j)(1-\psi_k)\chi_{jk}^{(aa)} + (1-\psi_j)\psi_k\chi_{jk}^{(ac)} + \psi_j(1-\psi_k)\chi_{jk}^{(ca)} + \psi_j\psi_k\chi_{jk}^{(cc)}\right] \Bigg] \\
= RT \Bigg[& \sum_{j\neq i}^{n} N_i \phi_j (1-\phi_i) \left[(1-\psi_i)(1-\psi_j)\chi_{ij}^{(aa)} + (1-\psi_i)\psi_j\chi_{ij}^{(ac)} + \psi_i(1-\psi_j)\chi_{ij}^{(ca)} + \psi_i\psi_j\chi_{ij}^{(cc)}\right] \\
& - \sum_{j\neq i}^{n} \sum_{\substack{k>j \\ k\neq i}}^{n} N_i \phi_j \phi_k \left[(1-\psi_j)(1-\psi_k)\chi_{jk}^{(aa)} + (1-\psi_j)\psi_k\chi_{jk}^{(ac)} + \psi_j(1-\psi_k)\chi_{jk}^{(ca)} + \psi_j\psi_k\chi_{jk}^{(cc)}\right] \Bigg] \\
= RTN_i \Bigg[& \sum_{j\neq i}^{n} \phi_j \Bigg((1-\phi_i) \left[(1-\psi_i)(1-\psi_j)\chi_{ij}^{(aa)} + (1-\psi_i)\psi_j\chi_{ij}^{(ac)} + \psi_i(1-\psi_j)\chi_{ij}^{(ca)} + \psi_i\psi_j\chi_{ij}^{(cc)}\right] \\
& - \sum_{\substack{k>j \\ k\neq i}}^{n} \phi_k \left[(1-\psi_j)(1-\psi_k)\chi_{jk}^{(aa)} + (1-\psi_j)\psi_k\chi_{jk}^{(ac)} + \psi_j(1-\psi_k)\chi_{jk}^{(ca)} + \psi_j\psi_k\chi_{jk}^{(cc)}\right] \Bigg) \Bigg] .
\end{aligned}
\tag{21}
$$

Eq. 16 is applied as well in this expression. Additionally, it can be remarked that the interaction parameters are considered here as constants. In cases where they bear an implicit dependency on the blend composition, further developments must be made. The three parts of the chemical potential can ultimately be summed together according to Eq. 13 to yield the relation presented in the core text.

D Parameters for Phase Diagram Calculations

Figure	N_1	N_2	v_0 [m^3/mol]	$\chi_{12}^{(aa)}$
2-a	100	1	1×10^{-5}	350/T
2-b	1	2	5×10^{-4}	2.1-180/T

Table 1: List of thermodynamic parameters used for the calculation of the phase diagrams presented in Fig.2 of the main article.

Figure	N_1	N_2	v_0 [m^3/mol]	$\chi_{12}^{(aa)}$	$\Delta\chi_{12}^{(ca)}$	Δh_1 [J/mol]	$T_{m,1}$ [K]	$\Delta\sigma_1$ [J/mol]
3-a	10	1	1×10^{-4}	0	100/T	-24000	550	48000
3-b	10	1	1×10^{-4}	0	0.2+350/T	-24000	550	48000
3-c	10	1	1×10^{-4}	0.3+110/T	0.2+350/T	-24000	550	48000
3-d	10	1	1×10^{-4}	0.4+250/T	0.2+350/T	-24000	550	48000

Table 2: List of thermodynamic parameters used for the calculation of the phase diagrams presented in Fig.3 of the main article.

Figure	N_1	N_2	v_0 [m^3/mol]	$\chi_{12}^{(aa)}$	$\Delta\chi_{12}^{(ca)}$	$\Delta\chi_{12}^{(ac)}$	$\Delta\chi_{12}^{(cc)}$
4-a	1	3	5×10^{-5}	0.3+150/T	0.05+50/T	0.1+100/T	0
4-b	1	3	5×10^{-5}	0.4+480/T	0.05+50/T	0.1+100/T	0
4-c	1	3	5×10^{-5}	0.3+150/T	0.05+50/T	0.1+100/T	0.3-30/T
4-d	1	3	5×10^{-5}	0.3+150/T	0.05+50/T	0.1+100/T	-60/T
Figure	Δh_1 [J/mol]	$T_{m,1}$ [K]	$\Delta\sigma_1$ [J/mol]	Δh_2 [J/mol]	$T_{m,2}$ [K]	$\Delta\sigma_2$ [J/mol]	
4-a	-900	500	1350	-4950	550	6600	
4-b	-900	500	1350	-4950	550	6600	
4-c	-900	500	1350	-4950	550	6600	
4-d	-900	500	1350	-4950	550	6600	

Table 3: List of thermodynamic parameters used for the calculation of the phase diagrams presented in Fig.4 of the main article.

Figure	N_1	N_2	N_3	v_0 [m^3/mol]	$\chi_{12}^{(aa)}$	$\chi_{13}^{(aa)}$	$\chi_{23}^{(aa)}$
5-a	50	50	1	1×10^{-5}	10/T	0	150/T
5-b	3	2	1	1×10^{-4}	0.3+300/T	0.3+400/T	0.4+500/T
5-c	100	10	1	1×10^{-5}	0.03+11/T	0.3+60/T	0.2-50/T
5-d	2	10	1	1×10^{-5}	0.15+100/T	0.5+280/T	0.3+350/T
5-e	5	15	1	1×10^{-5}	0.06+30/T	1.1	0.9
5-f	5	15	1	1×10^{-5}	0.06+30/T	0	0
Figure	$\Delta\chi_{12}^{(ca)}$	$\Delta\chi_{13}^{(ca)}$	$\Delta\chi_{12}^{(ac)}$	$\Delta\chi_{23}^{(ca)}$	$\Delta\chi_{12}^{(cc)}$	T [K]	
5-a	0	0	0	0	0	300	
5-b	0	0	0	0	0	300	
5-c	0.03+25/T	0.02	0	0	0	273	
5-d	0	0	0.0025+5/T	20/T	0	330	
5-e	0.01+10/T	0.02	0.02+20/T	0.05	0	330	
5-f	0.01+10/T	0.01	0.02+20/T	0.1	-0.2-50/T	650	
Figure	Δh_1 [J/mol]	$T_{m,1}$ [K]	$\Delta\sigma_1$ [J/mol]	Δh_2 [J/mol]	$T_{m,2}$ [K]	$\Delta\sigma_2$ [J/mol]	
5-a	0	0	0	0	0	0	
5-b	0	0	0	0	0	0	
5-c	-24000	550	48000	0	0	0	
5-d	0	0	0	-3600	500	4800	
5-e	-900	500	1350	-4950	550	6600	
5-f	-900	500	1350	-4950	550	6600	

Table 4: List of thermodynamic parameters used for the calculation of the phase diagrams presented in Fig.5 of the main article.

Figure	N_1	N_2	v_0 [m^3/mol]	$\chi_{12}^{(aa)}$	$\Delta\chi_{12}^{(ca)}$	Δh_1 [J/mol]	$T_{m,1}$ [K]	$\Delta\sigma_1$ [J/mol]
(SI) 1-a	5	1	1×10^{-4}	0.1+50/T	150/T	-10000	550	20000
(SI) 1-b	5	1	1×10^{-4}	0.3+400/T	100/T	-10000	550	20000
(SI) 1-c	5	1	1×10^{-4}	0.1+50/T	150/T	-10000	550	20000
(SI) 1-d	5	1	1×10^{-4}	0.3+400/T	100/T	-10000	550	20000

Table 5: List of thermodynamic parameters used for the calculation of the phase diagrams presented in Fig. 1 of the SI.

E Analysis of Free Energy Models basing on Landau Theory

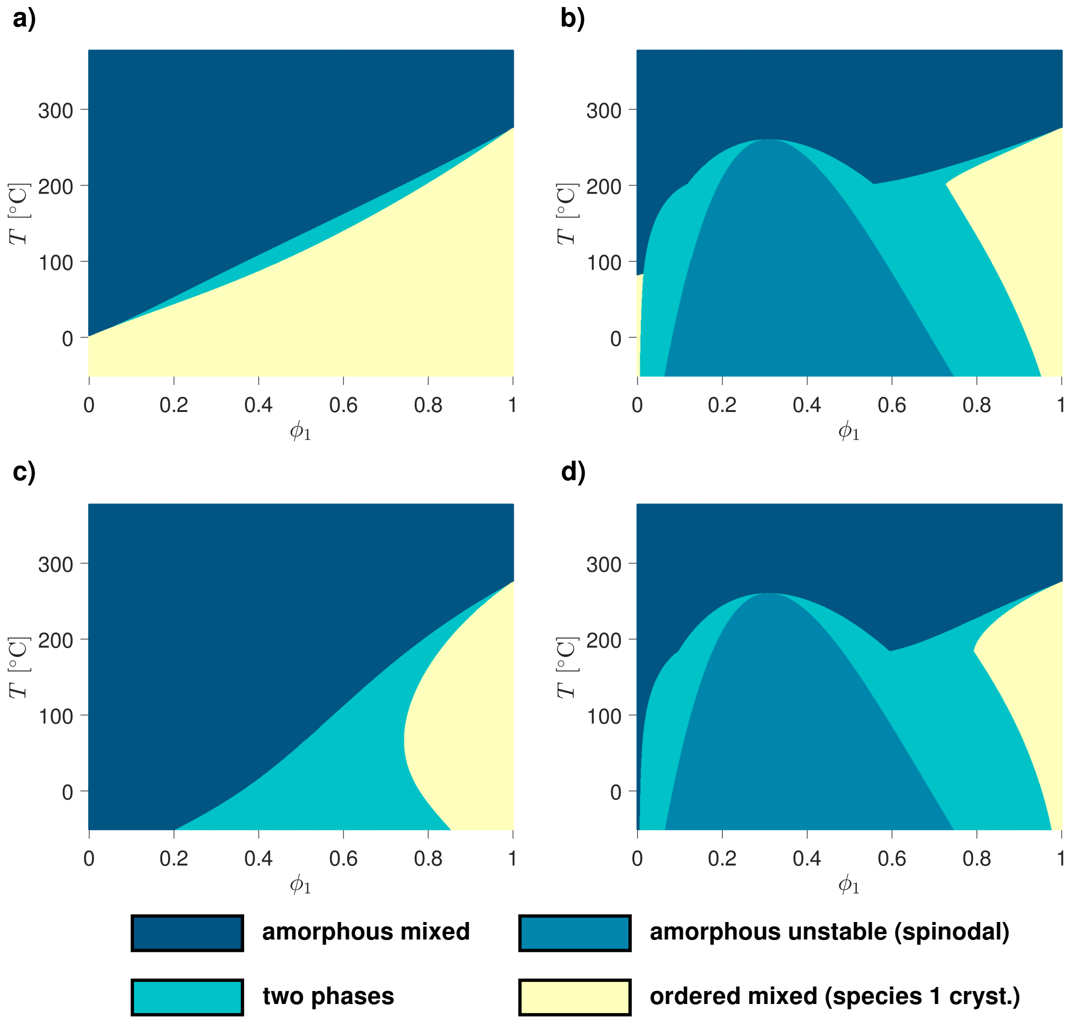

Figure 1: Comparison of binary phase diagrams produced from a)-b) the model of Matkar and Kyu [3][4] and c)-d) the framework derived in the present article. In all situations, solely the first blend constituent is able to undergo crystallization. The thermodynamic parameters used for the calculations are identical in a) and c), and b) and d), respectively (see Tab.5 in SI-D for the complete parameter sets). Below a given temperature (around 0°C in a) and 80°C in b)), it can be seen that the framework of Matkar and Kyu still predicts crystalline mixtures with however only trace amounts of this component (i.e. towards $\phi_1 = 0$). The exact location at which the threshold temperature is encountered depends on the strength of the crystalline-amorphous interactions, but is always existing. In contrast, the free energy formulation derived here generates diagrams exempt of this feature (the only case where the ordered state persists until $\phi_1 = 0$ is obtained in the limit where the incompatibility between the crystalline and the amorphous species vanishes, in other words when $\Delta\chi_{12}^{(ca)} = 0$).

As is detailed in Fig. 1, the model of Matkar and Kyu [3, 4] that describes crystallization processes with a Landau-type free energy possesses a shortcoming that is alleviated with the current formalism. The correction is mainly due to the prefactor ϕ_i^2 that multiplies the crystallization free energy density (instead of ϕ_i in the former framework [3, 4]). The Landau approach [5] is however also compatible with the free energy formula developed here. In this treatment, the free energy change triggered by a phase transition is expanded as a Taylor series. It introduces a state variable, the so-called "order parameter", which represents the degree of order of the studied system (typically, it ranges from 0, when the material is fully disordered, to 1 or, at least, non-zero values, when it is perfectly ordered). For first-order phase transitions such as crystallization, the resulting polynomial reads

$$\Delta G_i^{(c)} = C(T)\psi_i^2 - D(T)\psi_i^3 + E(T)\psi_i^4 + \mathcal{O}(\psi_i^5) \; , \tag{22}$$

where $C(T)$, $D(T)$, and $E(T)$ are positive coefficients that may be functions of the temperature T. $\Delta G_i^{(c)}$ refers to the crystallization free energy of species i. Its crystallinity ψ_i is regarded as the associated order parameter. One instance of Eq. 22 is considered per blend constituent. Other approaches may nonetheless be possible and would lead to developments different from those discussed hereafter. For instance, one might examine the crystallization energy of the whole mixture at once rather than keeping track of that of each component separately. The order parameter would then need to be adapted in consequence, and be required to reflect the effect of all crystallinities simultaneously.
It is now sought to identify the polynomial coefficients of Eq. 22 with the free energy terms responsible for the crystallization of species i in the main article, namely

$$\Delta G_i^{(c)} = \frac{n_0 N_A z}{2}\phi_i^2 \left[2\psi_i(1-\psi_i)\Delta G_{ii}^{(ac)} + \psi_i^2 \Delta G_{ii}^{(cc)}\right] \ . \tag{23}$$

In order to do so, it can be recognized that $\Delta G_{ii}^{(ac)}$ and $\Delta G_{ii}^{(cc)}$ must include further dependencies on ψ_i since the apparent polynomial degrees of both equations mismatch. For this, the definitions featured in the main text can be adjusted to

$$\begin{cases} \Delta G_{ii}^{(ac)} := q(\psi_i)\dfrac{\Delta\sigma_i}{N_i N_A z} \ , \\ \Delta G_{ii}^{(cc)} := p(\psi_i)\dfrac{2\Delta h_i}{N_i N_A z}\left(1-\dfrac{T}{T_{m,i}}\right) \ , \end{cases} \tag{24}$$

where $p(\psi_i)$ and $q(\psi_i)$ are also polynomials in ψ_i and the rest are constants. It can be verified that the following expressions for $p(\psi_i)$ and $q(\psi_i)$ satisfy the equation of the right hand sides of Eq. 22 and Eq. 23:

$$\begin{cases} q(\psi_i) = \psi_i(1-\psi_i) \ , \\ p(\psi_i) = 3 - 2\psi_i \ . \end{cases} \tag{25}$$

Indeed, replacing Eq. 24 and Eq. 25 into Eq. 23 and developing the resulting terms yields

$$\begin{aligned} \Delta G_i^{(c)} &= n_i\phi_i \left[\psi_i^2(1-\psi_i)^2\Delta\sigma_i + \psi_i^2(3-2\psi_i)\Delta h_i\left(1-\frac{T}{T_{m,i}}\right)\right] \\ &= \underbrace{n_i\phi_i\left[\Delta\sigma_i + 3\Delta h_i\left(1-\frac{T}{T_{m,i}}\right)\right]}_{C(T)}\psi_i^2 - \underbrace{2n_i\phi_i\left[\Delta\sigma_i + \Delta h_i\left(1-\frac{T}{T_{m,i}}\right)\right]}_{D(T)}\psi_i^3 + \underbrace{n_i\phi_i\Delta\sigma_i}_{E(T)}\psi_i^4 \ . \end{aligned} \tag{26}$$

The coefficients $C(T)$, $D(T)$, and $E(T)$ can thus be determined. Additionally, it can be remarked that Eq. 22 has to reduce to $C(T) - D(T) + E(T) = n_i\Delta h_i(1 - T/T_{m,i})$ in the case of a completely crystalline one-component system (i.e. $\phi_i = 1$ and $\psi_i = 1$). Keeping in mind that the molar latent heat of crystallization Δh_i bears a negative sign and is assumed to be constant, the following relationships can be deduced:

$$\begin{cases} D(T) = -n_i\Delta h_i \ , \\ C(T) + E(T) = -n_i\Delta h_i\dfrac{T}{T_{m,i}} \ . \end{cases} \tag{27}$$

Making use of the correspondences pointed out in Eq. 26, either equation can be rearranged to express $\Delta\sigma_i$ as a function of the latent heat Δh_i and the temperatures T and $T_{m,i}$:

$$\Delta\sigma_i = \left(\frac{T}{T_{m,i}} - \frac{3}{2}\right)\Delta h_i \ . \tag{28}$$

Moreover, since the coefficients of Eq. 22, especially $C(T)$, must be positive to ensure that the free energy takes a double-well shape suited for the modelling of phase transitions with energy barriers, it can be observed that $\Delta\sigma_i$ has to obey the inequality

$$\Delta\sigma_i \geq 3\Delta h_i\left(\frac{T}{T_{m,i}} - 1\right) \ . \tag{29}$$

Combining Eq. 28 and Eq. 29, one can find the expression of the critical temperature, sometimes referred to as "spinodal temperature" [6], below which the double-well shape and thus the energy barrier of Eq. 26 is no longer maintained:

$$T_{s,i} = \frac{3}{4}T_{m,i} \ . \tag{30}$$

Note that the constraint of a positive $E(T)$ (or $\Delta\sigma_i$) also implies a second critical temperature above $T_{m,i}$ at $T = (3/2)T_{m,i}$. In comparison, the model presented in the main manuscript (with $\Delta G_{ii}^{(ac)}$ and $\Delta G_{ii}^{(cc)}$ independent on ψ_i) is not restricted to this temperature interval. In this latter form, the crystallization free energy for species i writes

$$\Delta G_i^{(c)} = n_i\phi_i \left[\psi_i(1-\psi_i)\Delta\sigma_i + \psi_i^2\Delta h_i\left(1-\frac{T}{T_{m,i}}\right)\right] , \tag{31}$$

which is a second-degree polynomial in ψ_i. The abscissa ψ_i^* of its turning point (i.e. where the height of the energy barrier is maximal) is given by

$$\psi_i^* = \frac{\Delta\sigma_i}{2\left[\Delta\sigma_i - \Delta h_i\left(1-\frac{T}{T_{m,i}}\right)\right]} . \tag{32}$$

As long as $\Delta\sigma_i > 0$, ψ_i^* is also positive for any temperature below $T = T_{m,i}(1-\Delta\sigma_i/\Delta h_i)$. One may additionally constrain ψ_i^* to be comprised between 0 and 1, leading to a lowered limit temperature at $T = T_{m,i}(1-\Delta\sigma_i/2\Delta h_i)$. In any case, this maximum T is always higher than $T_{m,i}$ and, since $\Delta\sigma_i$ is here not explicitly related to Δh_i, the free energy formulation of Eq. 31 presents more flexibility than Eq. 26 regarding the temperature range on which it can be applied. Nevertheless, both formulae (Eq. 26 and Eq. 31) rely on the linearity approximation of the crystallization free energy (i.e. $\Delta h_i(1-T/T_{m,i})$) which is expected to be valid solely at relatively low degrees of undercooling.
Conversely, an advantage of the Landau-type free energy is its smooth polynomial double-well form, which is practical for extended formal analysis and may also be more convenient for numerical simulation purposes [6, 7]. It can further be remarked that Phase-field models based on the Landau free energy sometimes include an adjustable parameter that constrains the most energetically favorable value for ψ_i to be lower than 1 [3, 4, 8] (in other words, the global minimum of the double-well polynomial is shifted before $\psi_i = 1$, meaning that the system is not prone to undergo complete crystallization), so as to render phenomenologically the semi-crystallinity of polymers on macroscopic scales. However, this does not allow for modeling the mechanistic behavior of a partially crystalline material at the microscale. Further considerations are thus required in any of both frameworks to handle semi-crystallinity, either as a kinetic phenomenon (where amorphous portions of polymer chains elsewhere involved in crystal arrangements are hindered to displace freely, so that the crystallization process slows down significantly [9][10]), or thermodynamically through a modification of the free energy function (with multiple wells, for instance, in order to account for multistep ordering, or secondary recrystallization processes [11]).

References

1. Flory, P. J. *Principles of Polymer Chemistry* (Cornell University Press, 1953).
2. Rubinstein, M. & Colby, R. H. *Polymer Physics* (Oxford University Press, 2003).
3. Matkar, R. A. & Kyu, T. Phase Diagrams of Binary Crystalline-Crystalline Polymer Blends. *The Journal of Physical Chemistry B* **110,** 16059–16065 (2006).
4. Matkar, R. A. & Kyu, T. Role of Crystal-Amorphous Interaction in Phase Equilibria of Crystal-Amorphous Polymer Blends. *The Journal of Physical Chemistry B* **110,** 12728–12732 (2006).
5. Hohenberg, P. C. & Krekhov, A. P. An Introduction to the Ginzburg–Landau Theory of Phase Transitions and Nonequilibrium Patterns. *Physics Reports. An Introduction to the Ginzburg–Landau Theory of Phase Transitions and Nonequilibrium Patterns* **572,** 1–42 (2015).
6. Gránásy, L. *et al.* Phase-Field Modeling of Crystal Nucleation in Undercooled Liquids – A Review. *Progress in Materials Science* **106,** 100569 (2019).
7. Takaki, T. Phase-Field Modeling and Simulations of Dendrite Growth. *ISIJ International* **54,** 437–444 (2014).
8. Ronsin, O. J. J. & Harting, J. Phase-Field Simulations of the Morphology Formation in Evaporating Crystalline Multicomponent Films. *Advanced Theory and Simulations,* 2200286 (2022).
9. Menczel, J. D. The Rigid Amorphous Fraction in Semicrystalline Macromolecules. *Journal of Thermal Analysis and Calorimetry* **106,** 7–24 (2011).
10. Wunderlich, B. Termination of Crystallization or Ordering of Flexible, Linear Macromolecules. *Journal of Thermal Analysis and Calorimetry* **109,** 1117–1132 (2012).
11. Zhang, M. C., Guo, B.-H. & Xu, J. A Review on Polymer Crystallization Theories. *Crystals* **7,** 4 (2017).
